# A Multilayer Decision-Making Method for UAV Formation Cooperative Flight in Complex Urban Environments

**DOI:** 10.3390/s26103245

**Published:** 2026-05-20

**Authors:** Junjie Wang, Dongyu Yan, Yongping Hao, Han Miao

**Affiliations:** 1 School of Science, Shenyang Ligong University, Shenyang 110159, China; wjjsylu@sylu.edu.cn (J.W.); yandongyu1129@163.com (D.Y.); 15804013690@163.com (H.M.); 2 School of Equipment Engineering, Shenyang Ligong University, Shenyang 110159, China

**Keywords:** complex urban environment, virtual leader–follower, path planning, trajectory tracking, obstacle avoidance, formation cooperative flight

## Abstract

To address the challenges of dynamic obstacles, limited perception, and multi-UAV coordination constraints in complex urban environments, a hierarchical control framework based on a virtual leader-follower architecture is proposed, covering global planning, local obstacle avoidance, and formation coordination. In the global planning layer, a dynamic adaptive strategy rapidly exploring random tree star (DASRRT*) algorithm is proposed. To address the low sampling efficiency and limited path extension in dense environments that affect traditional RRT*, a hybrid guided sampling strategy, inefficient node optimization strategy, and perception-based adaptive step size strategy are designed. Additionally, a multi-objective cost function is introduced to provide smoother trajectories that better comply with dynamic constraints for trajectory tracking. In the local obstacle-avoidance layer, a distributed controller is constructed based on an improved artificial potential field method, integrating collision avoidance control laws derived from a spring-damper model, dynamic obstacle-avoidance laws that account for obstacle velocities, and formation coordination control laws grounded in consensus theory. In the coordination control layer, a real-time local target selection strategy is established to guide the virtual leader to precisely track the global path, and a dual-mode switching mechanism based on environmental complexity is constructed to dynamically adjust the priority between formation maintenance and autonomous obstacle-avoidance tasks. Comparative experimental results show that the proposed DASRRT* algorithm reduces path planning time by an average of 34.78% and shortens path length by 1.15%. Simulation results for formation flight demonstrate that the proposed hierarchical control framework can adaptively adjust control modes in response to changes in environmental complexity, exhibiting strong adaptability to complex environments and a good ability to generalize to various scenes.

## 1. Introduction

With the rapid development of unmanned aerial vehicle (UAV) technology, collaborative control strategies for UAV clusters have attracted significant attention [1,2]. Multi-UAV systems improve the intelligence and decision-making capability of individual agents through inter-agent coordination, offering advantages such as high flexibility, efficiency, low cost, and strong fault tolerance capability. Consequently, they are widely applied in both civil and military domains, including coordinated strikes [3,4,5], monitoring [6], logistics and transportation [7,8], and disaster relief [9]. To maintain an efficient formation configuration while ensuring safe mission execution, the system must be capable of rapid decision-making and accurate control under uncertain disturbances [10]. Although existing cooperative control theories have achieved substantial progress, traditional planning and control methods may fail when operating environments shift from open airspace to more complex and constrained scenarios.

With the rapid development of the low-altitude economy and smart cities, urban environments have become a core scenario for multi-UAV systems to execute missions. In applications such as civil urban air mobility and military low-altitude penetration, the safety and timeliness of UAV formations are crucial. However, urban airspace is characterized by significant unstructured and highly dynamic features. UAVs must maintain a compact formation to ensure cooperative effectiveness while also possessing high agility to avoid complex obstacles. Traditional methods struggle to balance the effectiveness of global path planning with the real-time requirements of local dynamic obstacle avoidance, often resulting in collisions or formation breakup when faced with sudden threats. Therefore, complex urban low-altitude formation tasks are inherently a “planning—control—coordination” coupling problem. The global layer needs to provide safe and feasible reference paths. The local layer must implement real-time obstacle and collision avoidance. The coordination layer must dynamically balance formation maintenance with autonomous obstacle avoidance and quickly restore the formation after obstacle avoidance. Thus, constructing a hierarchical cooperative framework that covers global planning, local obstacle avoidance, and formation coordination across three layers is an effective approach to improving the continuity and stability of formation tasks in complex urban environments.

Current multi-UAV path-planning methods mainly include graph-search methods, heuristic algorithms, deep reinforcement learning (DRL), and random sampling approaches [11,12,13,14,15]. Graph-search algorithms, such as A* and Dijkstra, can identify optimal paths on discretized grid maps. Reference [16] combined A* with the dynamic window approach and introduced an adaptive evaluation function to improve path quality; however, such methods incur high computational cost in complex or high-dimensional environments, and the resulting paths often lack smoothness. Heuristic algorithms, including genetic algorithms (GA) and ant colony optimization, address multi-constraint optimization problems by mimicking the behavior of biological populations. Reference [17] proposed a hierarchical framework based on an improved genetic algorithm and used time-dimensional path points for trajectory replanning, effectively resolving temporal conflicts in multi-UAV trajectories; nevertheless, such methods are prone to local optima and often exhibit limited solution stability. In recent years, DRL methods such as Proximal Policy Optimization and Deep Deterministic Policy Gradient (DDPG) have shown significant advantages in highly dynamic and unknown environments. Reference [18] introduces a multi-step smoothness metric as an auxiliary optimization objective, effectively suppressing trajectory oscillations by adaptively adjusting weight coefficients through an alternating hierarchical gradient descent strategy. However, DRL training is extremely time-consuming, and the resulting models lack interpretability. In addition, random sampling algorithms, such as RRT* and PRM, avoid full-map preprocessing by constructing path trees through spatial sampling, making them advantageous in high-dimensional spaces. The exploration-RRT* algorithm proposed in [19], for example, effectively balances information gain and travel cost in unknown environments. Nonetheless, such methods generally suffer from slow convergence and low sampling efficiency in narrow, constrained regions. The need to balance planning efficiency, path smoothness, and trajectory trackability in the constrained spaces of complex urban environments exacerbates the aforementioned shortcomings. Moreover, the global path must be capable of supporting subsequent path tracking and formation coordination control.

For cooperative formation flight control of multiple UAVs, efficient control strategies mainly include behavior-based methods, virtual structure methods, leader–follower methods, and artificial potential field (APF) approaches [20,21]. Behavior-based methods, inspired by biological swarm behavior, are highly distributed and well suited to large-scale swarms. Reference [22] simulated the flocking mechanism of starlings and achieved efficient formation control by using visual localization to resolve mutual perception. However, such methods generally lack rigorous mathematical guarantees for system stability and convergence. Virtual structure methods treat the formation as a rigid body and maintain the configuration by tracking virtual nodes. Reference [23] designed a sliding-mode tracker that computes tracking points according to the desired trajectory and terrain feature structure, thereby achieving high-precision formation maintenance. Nevertheless, the rigid constraints of this approach limit system flexibility, making it difficult to handle dynamic obstacle avoidance and formation reconfiguration in complex environments. Leader–follower methods maintain the formation by having followers track the leader’s state. Based on this framework, Reference [24] designed a three-channel robust controller to suppress dynamic uncertainties. Compared with the virtual structure approach, this method is more amenable to obstacle avoidance, but it suffers from the risk of single-point failure of the leader and lacks global coordination because the leader does not account for follower states. In addition, APF-based methods achieve formation control by constructing attractive fields toward the target and repulsive fields from obstacles. Reference [25] proposes an improved APF method that integrates predicted states and input thresholds, enhancing the control safety and real-time performance of large-scale swarm systems under delay constraints. However, in complex unstructured terrains such as three-point collinearity or semi-enclosed environments, APF is highly prone to local minima and may induce system oscillations. For urban constrained spaces, this means that formation control must not only be capable of obstacle avoidance but also require a control strategy that can adapt to different complex environments.

To address the inherent limitations of the above classical methods, extensive efforts have recently been devoted to targeted improvements. Although RRT* achieves asymptotic optimality through its rewiring mechanism, it still suffers from slow convergence and low sampling efficiency in narrow, constrained spaces [26,27]. To improve planning efficiency, Reference [28] proposed FRRT*, which integrates a risk network and a feedback module to constrain random-tree growth using situational information. Reference [29] further proposed the goal-biased PF-RRT*, which optimizes parent-node generation in obstacle neighborhoods through a bisection strategy, significantly reducing path cost and improving sampling quality. Traditional APF methods are prone to stagnation due to the balance between attractive and repulsive forces, and they may induce trajectory oscillations in complex terrains [30,31]. To address this issue, Reference [32] combined bionic swarm mechanisms with consensus theory and introduced a rotational potential function, effectively alleviating the local-minimum and oscillation problems in multi-UAV formations. In addition, to overcome the leader-failure problem in the leader–follower architecture, Reference [33] introduced a Virtual Leader combined with switching-topology communication, thereby effectively reducing computational burden. Reference [34] designed a finite-time consensus control law and incorporated an improved APF for dynamic obstacle avoidance, resolving the divergence of tracking errors in complex environments. Overall, existing research has made progress in areas such as planning efficiency, local obstacle avoidance, and consistency coordination. However, in complex urban scenarios, there is a need to further develop a hierarchical cooperative framework that can provide high-quality global reference trajectories while simultaneously ensuring formation maintenance, real-time obstacle avoidance, and formation recovery under local dynamic constraints.

Although multilayer decision-making and hierarchical control methods for UAVs have been widely studied, existing approaches mainly focus on one or two aspects, such as global path planning, local obstacle avoidance, formation maintenance, or constraint control. In contrast, this paper focuses on cooperative formation flight tasks in complex dynamic environments, with an emphasis on the collaborative design and improvement of global path planning, formation tracking control, and local obstacle avoidance. The main contributions of this paper are as follows:

(1) A dynamic adaptive strategy rapidly exploring random tree star (DASRRT*) algorithm is introduced. Unlike traditional RRT* and its variants, which primarily focus on search feasibility or path cost, DASRRT* combines guided sampling, adaptive step size, suboptimal node optimization, and path optimization strategies to improve search efficiency in complex, narrow environments while generating shorter, smoother global reference paths to enhance path traceability.
(2) A distributed control law based on an improved APF was designed. Compared with traditional APF or consensus control methods, this paper introduces a relative velocity damping term for collision avoidance and a target attraction factor and velocity adaptive weight for obstacle avoidance. Furthermore, a distributed formation cooperative control law based on consensus theory is developed to mitigate issues with traditional APF, such as local minima, oscillations, and insufficient response to dynamic obstacles. A stability analysis based on the Lyapunov method is also provided.
(3) A cooperative mechanism for formation control in complex environments is established. Unlike hierarchical control methods with fixed weights or a single mode, this paper establishes an environment complexity-based dual-mode switching and dynamic control weight adjustment method. This allows the formation to prioritize consistency in open areas, prioritize collision and obstacle avoidance in narrow or dynamically threatened regions, and quickly recover the desired formation once the risk is mitigated. In addition, a curvature-driven dynamic step size and local target selection strategy are designed, enabling the virtual leader to generate effective local guidance targets in real time along the global path.

## 2. Theoretical Foundation

This section introduces the fundamental concepts of algebraic graph theory, followed by the UAV dynamic model and its associated constraints. The content covered in this section pertains to the fundamental theory of cooperative control in multi-agent systems, with relevant definitions drawn from existing research on multi-agent consensus and graph theory-based control.

### 2.1. Graph Theory

**Figure 1:**
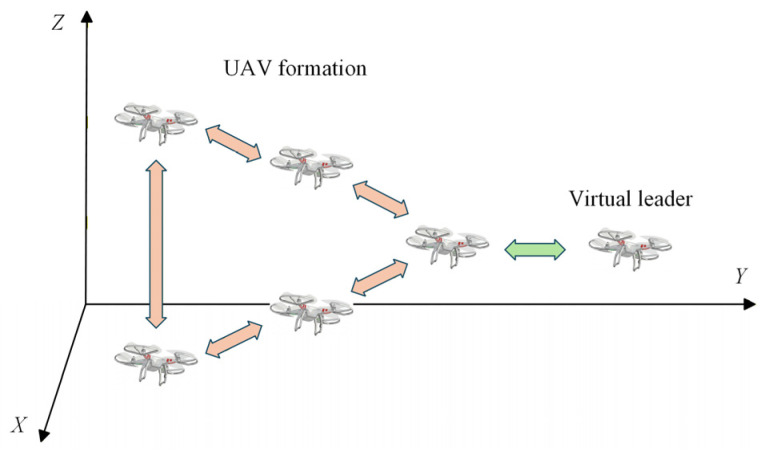
UAV formation topology.

Algebraic graph theory can be used to describe the information interaction among multiple UAVs and is widely applied in modeling UAV formation cooperative control [35,36]. The considered formation system consists of one virtual leader and n followers, where an undirected graph G represents the communication topology among followers, as illustrated in Figure 1. During formation flight, each UAV is assumed to receive the leader’s position and velocity information without delay or packet loss. In addition, followers can access information from neighboring agents only when they are within the prescribed communication range.

The graph $G=\left(V,E\right)$ consists of a node set $V=\left\{1,2,\cdots ,n\right\}$ and an edge set $E=\left\{\left(i,j\right)|i,j\in V,i\neq j\right\}$. For the i-th follower, the set of its neighboring UAVs is defined as $N_{i}=\left\{j\in V,\left(i,j\right)\in E\right\}$. Let A denote the adjacency matrix of G, where $a_{ij}$ represents the weight of the edge $\left(i,j\right)$. Defining the communication topology as an undirected graph implies that the i-th UAV and the j-th UAV can mutually exchange state information. Specifically, if $\left(i,j\right)\in E$, then $a_{ij}=1$; otherwise, $a_{ij}=0$. It is further assumed that the graph contains no self-loops, such that $a_{ii}=0$. The degree matrix is given by $D=diag\left\{d_{1},d_{2},\cdots {,d}_{n}\right\}$, where $d_{i}=\sum_{j=1,j\neq i}^{n}a_{ij}$, and the corresponding Laplacian matrix is defined as L=D−A. In the context of this leader–follower multi-UAV system, the leader is designated as UAV 0. In the virtual leader–follower multi-UAV system, the virtual leader UAV is denoted as UAV 0. Let $H\in R^{n\times n}$ represent the connection matrix between the followers and the leader, defined as $H=diag(h_{1},h_{2},\ldots ,h_{n}).$ If the i-th follower exchanges information with the leader, then $h_{i}=1$; otherwise, $h_{i}=0$.

### 2.2. Models and Constraints

#### 2.2.1. UAV Dynamics Model

To account for aerodynamic drag and inertial effects during actual flight, the i-th UAV is modeled as a second-order point mass dynamical system with damping characteristics [37,38]. Let $p_{i}(t)={\left[x_{i},y_{i},z_{i}\right]}^{T}\in R^{3}$ and $v_{i}(t)={\left[v_{ix},v_{iy},v_{iz}\right]}^{T}\in R^{3}$ denote the position and velocity vectors of the UAV at time t, respectively.

(1) $$\left\{\begin{array}{c} \dot{p_{i}}\left(t\right)=v_{i}\left(t\right)\\ \dot{v_{i}}\left(t\right)=\dot{u_{i}}\left(t\right)-\gamma \dot{v_{i}}\left(t\right) \end{array}\right.$$

where $u_{i}\left(t\right)\in R^{3}$ represents the control input vector, and γ>0 denotes the air damping coefficient, which reflects the energy dissipation characteristics of the actual flight environment. Given a sampling time step Δt, the state update equations at time t+1 are formulated as follows:

(2) $$\left\{\begin{array}{c} v_{i}\left(t+1\right)=\left(1-\gamma \Delta t\right)v_{i}\left(t\right)+u_{i}\left(t\right)\Delta t\\ p_{i}\left(t+1\right)=p_{i}\left(t\right)+v_{i}\left(t+1\right)\Delta t \end{array}\right.$$

#### 2.2.2. UAV Constraints

In practical applications, the maneuverability of a UAV is restricted by its physical actuators and must adhere to safe flight constraints [39]. This research incorporates the following three categories of constraints:

(1) Saturation Constraints

To ensure that control commands remain within the feasible operational range of the actuators, saturation constraints are imposed on flight velocity and control input magnitude.

Specifically, the flight speed of a UAV must not exceed the maximum allowable velocity $v_{max}$.

(3) $$\left\|v_{i}\left(t\right)\right\|\leq v_{max},\;\;\forall i\in n$$

Furthermore, the control input is bounded by the maximum acceleration $u_{max}$.

(4) $$\left\|u_{i}\left(t\right)\right\|\leq u_{max},\;\;\forall i\in n$$

(2) Obstacle Avoidance Safety Constraints

The flight environment comprises a set of static obstacles $O_{s}$ and dynamic obstacles $O_{d}$. Let $\Omega_{k}$ denote the geometric region occupied by the k-th obstacle. To guarantee flight safety, the distance between a UAV and any obstacle must be maintained above a safety margin $\delta_{safe}$.

(5) $$\underset{o\in \Omega_{k}}{\min}\left\|p_{i}\left(t\right)-p_{o}\right\|\geq \delta_{safe},\;\;\forall k\in O_{s}\cup O_{d}$$

A collision is considered to have occurred whenever the distance between the UAV and an obstacle is less than this safety margin.

(3) Inter-agent Collision Avoidance Constraints

To prevent internal collisions within the formation, the distance between any two UAVs must consistently remain greater than a minimum safety interval $r_{s}$.

(6) $$\left\|p_{i}\left(t\right)-p_{j}\left(t\right)\right\|>r_{s},\;\;\forall i,j\in n,\;i\neq j$$

A collision is deemed to have occurred if the distance between two UAVs falls below this safety interval.

## 3. Methodology

**Figure 2:**
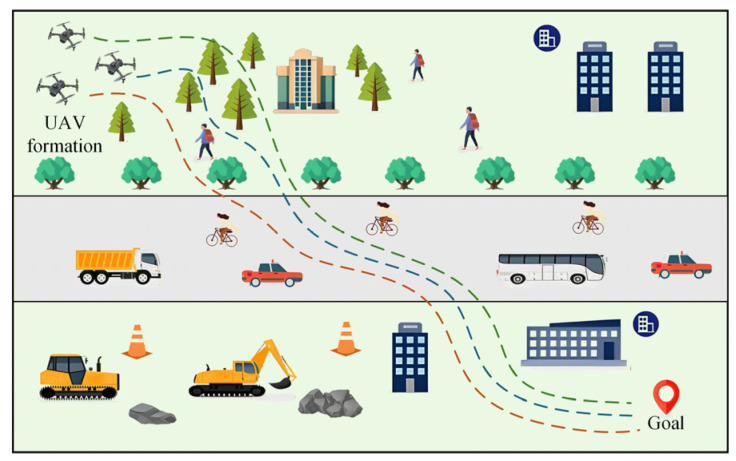
Urban flight scenario diagram.

This study introduces a novel hierarchical decision and control framework based on the virtual leader–follower strategy, designed to collaboratively resolve the challenges of global path planning, local obstacle avoidance, and formation cooperative control for UAVs within complex dynamic environments. The operational flight scenario in a complex environment is shown in Figure 2.

### 3.1. Global Path Planning

**Figure 3:**
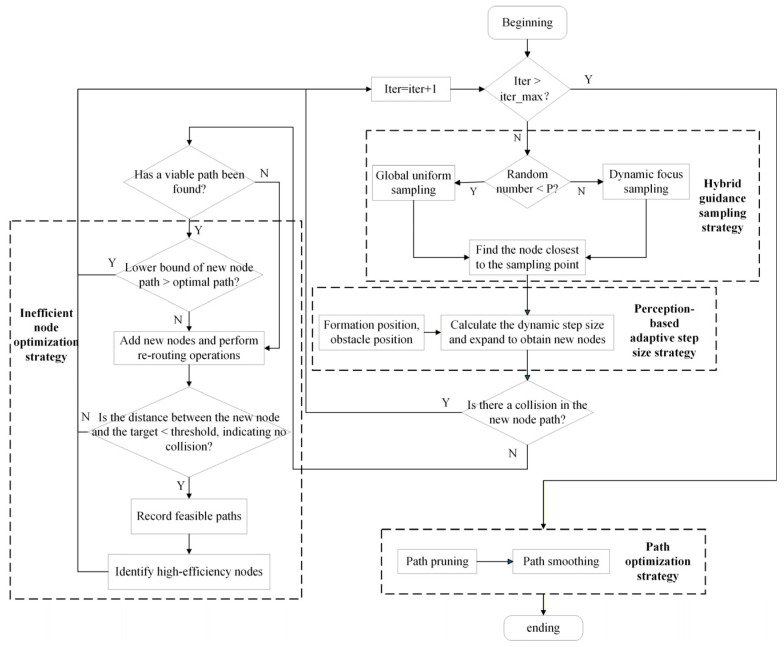
Framework of the DASRRT* algorithm.

To generate high-quality global reference paths, the DASRRT* algorithm is developed. Building upon the traditional RRT, it integrates four synergistic optimization strategies to improve planning efficiency and path quality in narrow and complex environments. The overall framework of DASRRT* is illustrated in Figure 3.

#### 3.1.1. Hybrid Guided Sampling Strategy

To improve the low sampling efficiency of traditional RRT algorithms, a hybrid guided sampling mechanism is introduced. At each iteration, dynamic focused sampling is selected with probability $P_{f}$, while global uniform sampling is applied with probability ${1-P}_{f}$. The dynamic focused strategy guides the sampling point $q_{rand}$ within a concentric spherical region that adapts according to the iteration index k.

(7) $$q_{rand}=\left\{\begin{array}{c} \text{dynamic}\;\text{focused}\;\text{sampling},\;\text{with}\;\text{probability}\;P_{f}\\ \text{global}\;\text{uniform }\;\text{sampling},\;\text{with}\;\text{probability}\;1-P_{f} \end{array}\right.$$

where $q_{rand}$ denotes the geometrically guided sampling point, and $P_{f}$ represents the sampling bias weight.

(1) Global Uniform Sampling

Uniformly distributed sampling points $q_{rand}$ are generated within the map space as follows:

(8) $$q_{rand}=\left[\begin{array}{c} x_{rand}\\ y_{rand}\\ z_{rand} \end{array}\right],\;\;\left\{\begin{array}{c} x_{rand}\in \left[x_{min},x_{max}\right]\\ \begin{array}{c} y_{rand}\in \left[y_{min},y_{max}\right]\\ z_{rand}\in \left[z_{min},z_{max}\right] \end{array} \end{array}\right.$$

where $\left[x_{min},x_{max}\right]\times \left[y_{min},y_{max}\right]\times \left[z_{min},z_{max}\right]$ defines the environmental boundaries.

(2) Dynamic Focused Sampling

**Figure 4:**
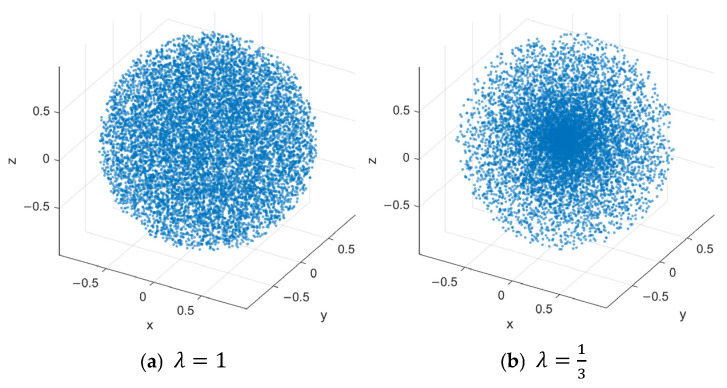
Sampling distributions for two different values of λ.

The center of the focused region, $q_{c}$ is adaptively adjusted via a bias weight $P_{g}$.

(9) $$q_{c}=\left\{\begin{array}{c} q_{goal},\;\;\text{with}\;\text{probability}\;P_{g}\\ \frac{q_{start}+q_{goal}}{2},\;\;\text{with}\;\text{probability}\;1-P_{g} \end{array}\right.$$

where $q_{start}$ denotes the starting point, $q_{goal}$ is the target point, and $P_{g}$ represents the target bias weight. Furthermore, the radius of the focused region, r(k), evolves dynamically as a function of the iteration index k and the environmental scale $\left\|q_{goal}-q_{start}\right\|$.

(10) $$r\left(k\right)=\rho \left\|q_{goal}-q_{start}\right\|{\left(\frac{i_{k}}{k}\right)}^{\lambda }$$

where, ρ is the scale factor, $i_{k}∼U(0,1)$ is a random variable, and λ is the shape parameter controlling the concentration of the sampling distribution. When $\lambda =\frac{1}{3}$, sampling is uniformly distributed within the sphere; for $\lambda >\frac{1}{3}$, sampling points concentrate toward the sphere center. For representative cases $\lambda =\frac{1}{3}$ and λ=1, the distribution of 10,000 samples within a unit sphere is shown in Figure 4.

Once the sampling center and radius are determined, sampling points are randomly generated within the spherical domain as follows:

(11) $$q_{rand}=q_{c}+r\left(k\right)v_{\theta },\;\;v_{\theta }={\left(sin\phi cos\theta ,sin\phi sin\theta ,cos\phi \right)}^{T}$$

where, $\left.\theta ∼U[0,2\pi \right)$ denotes the azimuth angle, and ϕ=arccos(2η−1) denotes the polar angle, where η∼U[0,1]. This formulation ensures uniform sampling within the corresponding spherical region in 3D space.

To ensure that the hybrid guided sampling point $q_{rand}$ remains within the environmental boundary $B=\left[x_{min},x_{max}\right]\times \left[y_{min},y_{max}\right]\times \left[z_{min},z_{max}\right]$, coordinate-wise boundary constraints are enforced as follows:

(12) $$q_{rand}^{\left(i\right)}=max\left(min\left(q_{rand}^{\left(i\right)},B_{max}^{\left(i\right)}\right),B_{min}^{\left(i\right)}\right),\;\;\;i\in \left\{x,y,z\right\}$$

When $\lambda >\frac{1}{3}$, dynamic focused sampling yields an adaptive distribution that is dense near the center and sparse near the boundary, accelerating exploration of the target region and facilitating rapid discovery of an initial feasible path.

#### 3.1.2. Perception-Based Adaptive Step Size Strategy

**Figure 5:**
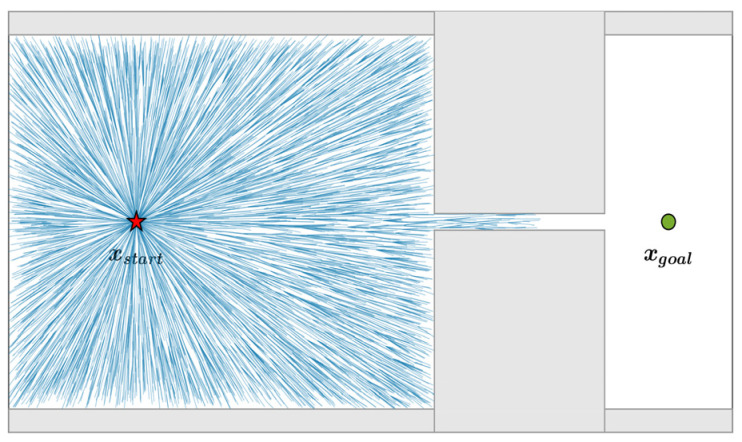
Limitations in narrow passages.

The limitations of conventional RRT* in narrow passages are shown in Figure 5. Owing to the stochastic nature of sampling and the use of a fixed expansion step size, the effective expansion rate remains low in obstacle-dense regions. When nodes approach obstacles, an excessively large step size often causes newly generated nodes to collide with obstacles, resulting in invalid expansions. In contrast, an overly small step size reduces pathfinding efficiency.

To prevent expansion failures caused by excessively large step sizes near obstacles, a dynamic obstacle-aware step size strategy is proposed. The expansion step size δ is adaptively adjusted according to the minimum Euclidean distance $d_{obs}$ from the neighboring node $q_{near}$ to the closest obstacle.

(13) $$\delta \left(q\right)=max\left(\delta_{max}\left(1-e^{-\beta d_{obs}\left(q_{near}\right)}\right),\delta_{min}\right)$$

where, $\delta_{max}$ and $\delta_{min}$ denote the upper and lower bounds of the step size, respectively; $d_{obs}(q_{near})$ is the distance from the node to the nearest obstacle; and β∈(0,1) is a sensitivity parameter. When $d_{obs}$ is small, indicating proximity to obstacles or narrow passages, δ decreases to improve expansion precision and feasibility. Conversely, in open regions, a larger step size is maintained to accelerate exploration.

#### 3.1.3. Inefficient Node Optimization Strategy

To alleviate the rapid growth in computational cost caused by neighborhood search and rewiring during tree expansion, an optimization mechanism for the rewiring process is introduced. After identifying an initial feasible path, each newly generated node $q_{i}$ is evaluated using:

(14) $$\Phi \left(q_{i}\right)=\left\|q_{i}-q_{start}\right\|+\left\|q_{i}-q_{goal}\right\|$$

The set satisfying $\Phi (q_{i})=c_{best}$ forms an ellipsoidal surface with $q_{start}$ and $q_{goal}$ as the foci. Nodes within or on this ellipsoid are considered efficient. If $\Phi (q_{i})>c_{best}$, any path passing through $q_{i}$ will exceed the current optimal path length, as $\Phi (q_{i})$ provides a lower bound on feasible path length via $q_{i}$. Therefore, such nodes are discarded to reduce memory usage and computational overhead.

Moreover, whenever the optimal path is updated, all existing nodes are re-evaluated. Nodes satisfying $\Phi (q_{i})>c_{best}$ are labeled as inefficient and excluded from subsequent nearest-neighbor and near-neighbor searches.

To further analyze the computational complexity of DASRRT*, the algorithm’s final node count is denoted as $N_{\mathrm{node}}$, and the average obstacle detection value is represented as $C_{\mathrm{col}}$. The number of nodes in the i-th iteration of the search tree is denoted as $N_{i}$, and the number of nodes in the neighboring search area is $k_{i}$. For the base RRT*, each iteration requires searching in the current node set and nearby nodes, where the number of nodes is related to $N_{i}$; similarly, the parent nodes need to choose and re-connect to the nearby nodes to perform collision detection. Therefore, the total computational complexity of the base RRT* is expressed as:

(15) $$T_{RRT*}=O\left(\sum_{i=1}^{N_{node}}\left(N_{i}+k_{i}C_{col}\right)\right)$$

where:

(16) $$\sum_{i=1}^{N_{node}}N_{i}=1+2+\cdots +N_{node}=\frac{N_{node}\left(N_{node}+1\right)}{2}$$

Under normal conditions, $k_{i}<N_{i}$, and since the collision detection in each iteration is constant, the time complexity of RRT* is approximately:

(17) $$T_{RRT*}=O\left({N_{node}}^{2}\right)$$

DASRRT* incorporates low-efficiency node optimization into the base algorithm. In the i-th iteration, the number of effective nodes is denoted as $N_{i}^{a}$, where $N_{i}^{a}\leq N_{i}$. Ineffective nodes are no longer considered in the subsequent search or nearby search operations. Therefore, the actual search complexity of DASRRT* can be expressed as:

(18) $$T_{DASRRT*}=O\left(\sum_{i=1}^{N_{node}}\left(N_{i}^{a}+k_{i}^{a}C_{col}\right)+\sum_{u=1}^{U}N_{u}\right)$$

where U represents the number of times the optimal path has been updated, and $N_{u}$ represents the number of nodes updated each time. Compared with RRT*, DASRRT* updates the nodes of the optimal path after each update and then performs low-efficiency node pruning. Since the number of optimal path updates is typically much smaller than the total number of iterations, this term does not dominate the overall complexity in practice. Let the proportion of valid nodes be:

(19) $$\alpha_{i}=\frac{N_{i}^{a}}{N_{i}},\;\;\;0<\alpha_{i}<1$$

The actual search complexity of DASRRT* is approximated as:

(20) $$T_{DASRRT*}=O\left({\alpha_{i}N_{node}}^{2}\right)$$

From a strict asymptotic perspective, if $\alpha_{i}$ is considered constant, the worst-case time complexity of DASRRT* remains $O(N_{no\mathrm{de}}^{2})$. Inefficient node optimization primarily reduces the node size and complexity coefficient in the actual search, thereby decreasing the computational overhead to some extent.

**Figure 6:**
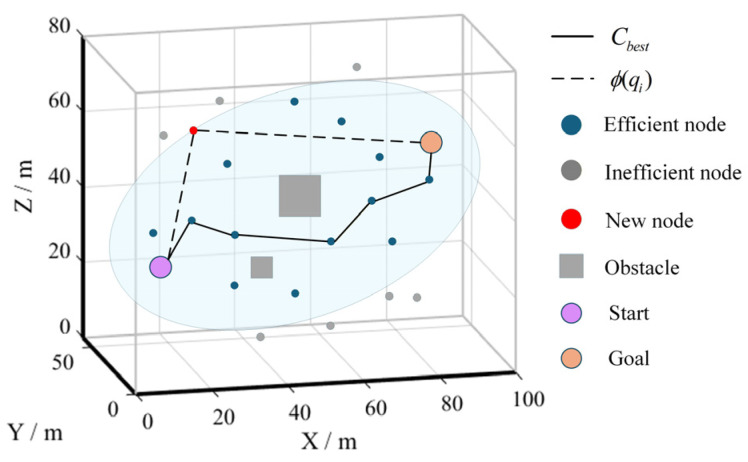
Schematic of the inefficient node optimization strategy.

The implementation of the inefficient node optimization strategy is shown in Figure 6. By pre-screening and eliminating inefficient nodes, the total number of nodes is reduced, thereby improving computational efficiency. Since these nodes no longer participate in subsequent expansions, the growth of the tree is restrained, leading to improved memory utilization.

#### 3.1.4. Path Optimization Strategy

##### Path Pruning

Utilizing the maximum leap method, an initial path $\pi =\{q_{1},q_{2},\ldots ,q_{N}\}$ is pruned by traversing backward from the end node $q_{N}$ for each node $q_{i}$ to identify the furthest collision-free node $q_{j}(j>i)$ for direct connection, thereby significantly shortening the total path length. The optimized path π is defined as follows:

(21) $$\pi =\overset{N}{\underset{i=1}{⋃}}q_{i}$$

where $q_{i+1}=max\{j\in (i,N]|C(q_{i},q_{j})=\mathrm{True}\}$. $C(q_{i},q_{j})=\mathrm{True}$ indicates that the straight-line segment connecting $q_{i}$ and $q_{j}$ is collision-free.

The specific steps are as follows:

Step 1: Initialization. Set $\pi =\{q_{1}\}$ and initialize the current index i=1.

Step 2: Backward Search. Traverse backward from the end point $q_{N}$ to find the furthest node $q_{j}(j>i)$ that satisfies $C(q_{i},q_{j})=\mathrm{True}$.

Step 3: Path Update. Append $q_{j}$ to π and update the current index i=j.

Step 4: Termination. Repeat Steps 2 and 3 until $q_{j}=q_{N}$.

**Figure 7:**
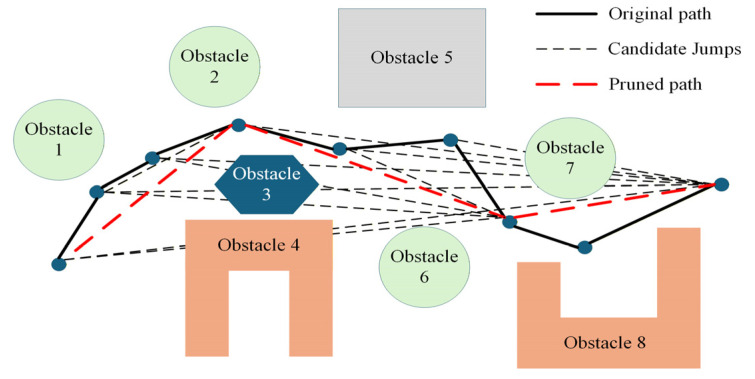
Schematic diagram of path pruning.

The aforementioned path pruning operation selects the maximum feasible leap at each step, ensuring that the total path length decreases monotonically and that optimality is effectively improved, as illustrated in Figure 7.

##### Path Smoothing

To mitigate the issue of sharp turns within the pruned path, an iterative path smoothing algorithm is proposed. By optimizing path continuity and smoothness, this method effectively enhances the path-tracking performance of the UAV formation.

(1) Objective Function Design

Given a set of discrete, collision-free path points after pruning, $\pi =\{q_{1},q_{2},\ldots ,q_{N}\}$, the objective is to solve for a new trajectory that ensures both smoothness and obstacle avoidance.

To guarantee trajectory smoothness, the second-order difference of path points—representing discrete acceleration—is minimized. For a sequence of discrete points, this second-order difference is approximated as $\Delta^{2}q_{i}=q_{i-1}-2q_{i}+q_{i+1}$. Accordingly, the objective function of the smoothing term is defined as follows:

(22) $$J_{s}\left(\pi \right)=\sum_{i=1}^{N}{\left\|q_{i-1}-2q_{i}+q_{i+1}\right\|}^{2}$$

To prevent over-smoothing that might cause the trajectory to penetrate obstacles, a penalty is imposed on the distance between the optimized point $q_{i}^{new}$ and the original reference point $q_{i}$. Accordingly, the objective function of the penalty term is defined as follows:

(23) $$J_{p}\left(\pi \right)=\sum_{i=1}^{N}w_{i}{\left\|q_{i}-q_{i}^{new}\right\|}^{2}$$

The final composite objective function to be minimized is formulated as follows:

(24) $$J\left(\pi \right)=\lambda_{s}J_{s}\left(\pi \right)+\lambda_{p}J_{p}\left(\pi \right)$$

(2) Solution Methodology

To facilitate an efficient solution, the aforementioned summation is reformulated into a matrix representation. Let $\pi =[q_{1},q_{2},\ldots ,q_{N}{]}^{T}$ denote the trajectory matrix to be determined.

For the smoothness term, the second-order difference operator can be expressed in the following tridiagonal matrix form:

(25) $$A=\frac{1}{h^{2}}{\left[\begin{array}{ccccc} 2 & -1 & 0 & \ldots & 0\\ -1 & 2 & -1 & \ddots & \vdots \\ 0 & -1 & 2 & \ddots & 0\\ \vdots & \ddots & \ddots & \ddots & -1\\ 0 & \cdots & 0 & -1 & 2 \end{array}\right]}_{n\times n}$$

where h denotes the spatial discretization step size, N denotes the number of path points, and n=N−2 (as the start and end points of the path cannot serve as intermediate nodes for acceleration calculations). Accordingly, the smoothness objective function is expressed as follows:

(26) $$J_{s}=\pi^{T}A^{T}A\pi$$

For the distance penalty term, the weight assigned to each path point is represented in the following matrix form:

(27) $$W={\left[\begin{array}{ccccc} w_{1} & 0 & 0 & \ldots & 0\\ 0 & w_{2} & 0 & \ddots & \vdots \\ 0 & 0 & w_{3} & \ddots & 0\\ \vdots & \ddots & \ddots & \ddots & 0\\ 0 & \ldots & 0 & 0 & w_{n} \end{array}\right]}_{n\times n}$$

where $w_{i}$ denotes the distance deviation weight for the i-th path point. Let the updated path trajectory be denoted by $\pi^{\mathrm{new}}$. The objective function for the distance deviation is calculated as follows:

(28) $$J_{p}={\left(\pi -\pi^{new}\right)}^{T}W{\left(\pi -\pi^{new}\right)}^{T}$$

To obtain the optimal trajectory, we seek π such that ∇J(π)=0. Expanding the total cost function and differentiating with respect to π yields:

(29) $$\frac{\partial J}{\partial \pi }=\lambda_{S}A^{T}A\pi +\lambda_{P}W\left(\pi -\pi^{new}\right)=0$$

Rearranging the terms results in the following system of linear equations:

(30) $$\left(\lambda_{S}A^{T}A+\lambda_{P}W\right)\pi =\lambda_{P}W\pi^{new}$$

The final solution for π minimizes the combined sum of the second-order differences and the distance deviations of the path points.

(3) Solution Procedure

The iterative procedure for smoothing the initial path is summarized as follows:

Step 1: Initialization. Assign initial values to the weights $w_{i}$ for all path points.

Step 2: Optimization. Solve the system of linear equations based on the current weight matrix W to obtain the trajectory $\pi^{\mathrm{new}}$.

Step 3: Collision detection. Traverse each point in $\pi^{\mathrm{new}}$ to determine if it resides within any obstacle O. Points found to be in collision are added to the set C.

Step 4: Weight update. If C is non-empty, increase the distance deviation penalty weights for the colliding nodes and their neighboring points.

Step 5: Termination condition. Repeat Steps 2 through 4 until C=∅ or the maximum number of iterations is reached.

### 3.2. Local Cooperative Control

**Figure 8:**
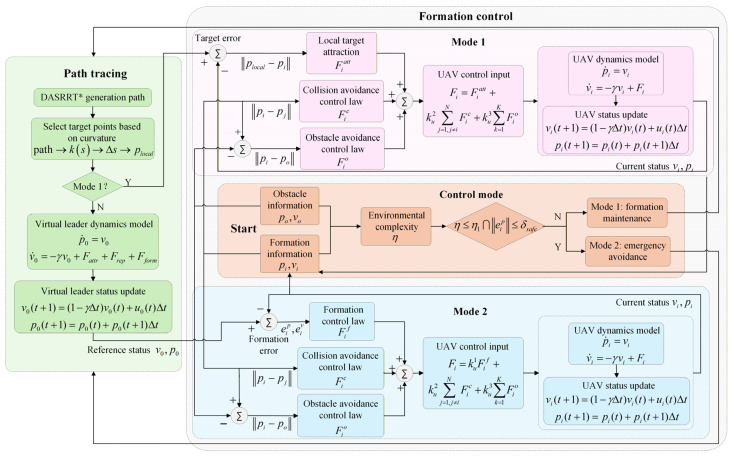
Formation flight control framework.

This section develops internal collision avoidance, external obstacle avoidance, and formation cooperative control laws. Under the governance of these control laws, each UAV tracks the virtual leader. The integrated formation flight control architecture is illustrated in Figure 8.

#### 3.2.1. Collision-Avoidance Control Law

To prevent collisions caused by close proximity and velocity differences among UAVs during formation flight, a collision-avoidance control law is designed. By introducing a relative-velocity damping term on the basis of the conventional distance-based repulsive force, the proposed method suppresses oscillations while maintaining a safe separation distance, thereby improving stability during dense formation maneuvers.

The repulsive potential energy is defined as:

(31) $$U_{c}\left(\left\|p_{ji}\right\|\right)=\frac{1}{2}\frac{k_{1}^{c}}{{\left(\left\|p_{ji}\right\|-r_{s}\right)}^{2}}$$

The repulsive force exerted by the j-th UAV on the i-th UAV is derived as:

(32) $$F_{ji}^{c}=-\nabla_{p_{i}}U_{c}=-\frac{dU_{c}}{d\left\|p_{ji}\right\|}\frac{d\left\|p_{ji}\right\|}{dp_{i}}=-\frac{k_{1}^{c}}{{\left(\left\|p_{ji}\right\|-r_{s}\right)}^{3}}\frac{p_{ji}}{\left\|p_{ji}\right\|}$$

Consequently, the total collision-avoidance force $F_{i}^{c}$ acting on the i-th UAV is given by:

(33) $$F_{i}^{c}=\sum_{j=1,j\neq i}^{N}\left(-\frac{k_{1}^{c}}{{\left(\left\|p_{ji}-r_{s}\right\|\right)}^{3}}\frac{p_{ji}}{\left\|p_{ji}\right\|}-k_{2}^{c}v_{ji}\right)$$

where $k_{1}^{c},k_{2}^{c}$ denote the repulsive gain coefficients, which regulate the influence of the position error and velocity damping terms, respectively. $p_{ji}$ denotes the position difference vector between UAV j and i, and $v_{ji}$ denotes their relative velocity difference vector. $r_{s}$ denotes the safety distance of the UAV formation. In the expression, the first term represents the positional repulsion responsible for maintaining inter-UAV distances, while the second term provides velocity damping to effectively suppress relative velocities and minimize oscillations.

#### 3.2.2. Obstacle-Avoidance Control Law

To enhance the obstacle-avoidance capability of the formation in complex environments with dynamic obstacles, an obstacle-avoidance control law is designed. Based on the artificial potential field method, the proposed law incorporates a goal-attraction factor and a velocity-adaptive mechanism, effectively alleviating the problems of target inaccessibility, local stagnation, and insufficient responsiveness to dynamic obstacles.

The repulsive potential energy exerted by obstacle o on UAV i is defined as:

(34) $$U_{i}^{o}=\left\{\begin{array}{c} \frac{1}{2}k_{1}^{o}{\left(\frac{1}{\left\|p_{io}\right\|}-\frac{1}{r_{s}}\right)}^{2}{\left\|p_{ig}\right\|}^{n},\;\;\;0<\left\|p_{io}\right\|\leq r_{u}\\ 0,\;\;\;\;\left\|p_{io}\right\|>r_{u} \end{array}\right.$$

where $p_{io}$ denotes the distance from UAV i to obstacle o, $r_{u}$ denotes the detection radius of the UAV, and $p_{ig}$ denotes the distance from UAV i to the goal point g.

(1) Target Attraction Factor

By introducing the term ${\left\|p_{ig}\right\|}^{n}$, the repulsive force decays concurrently with the attractive force as the UAV approaches the goal ($\left\|p_{ig}\right\|\to 0$). Upon reaching the destination, the repulsive force becomes zero, effectively resolving the issues of target unreachability and local stagnation.

(2) Velocity-Adaptive Weighting

(35) $$k_{1}^{o}=k_{2}^{o}\left(1+k_{3}^{o}e^{\frac{-1}{1+v_{k}^{o}}}\right)$$

where $k_{2}^{o}$ and $k_{3}^{o}$ are weight adjustment parameters. The gain $k_{1}^{o}$ is coupled with the obstacle’s velocity; a higher obstacle velocity $v_{k}^{o}$ results in a larger gain $k_{1}^{o}$, enabling a rapid response to high-speed dynamic obstacles.

Based on the modified repulsive potential function, the corresponding obstacle-avoidance control force can be derived as follows:

(36) $$F_{i}^{of}=-\nabla_{p_{i}}U_{i}^{o}=\frac{\partial U_{i}^{o}}{\partial \left\|p_{io}\right\|}\frac{\partial \left\|p_{io}\right\|}{\partial p_{i}}+\frac{\partial U_{i}^{o}}{\partial \left\|p_{ig}\right\|}\frac{\partial \left\|p_{ig}\right\|}{\partial p_{i}}$$

To ensure system stability, a velocity damping term is incorporated:

(37) $$F_{i}^{ov}=-k_{4}^{o}v_{i}$$

The total control force for the i-th UAV is then obtained as:

(38) $$F_{i}^{o}=F_{i}^{of}+F_{i}^{ov}$$

**Figure 9:**
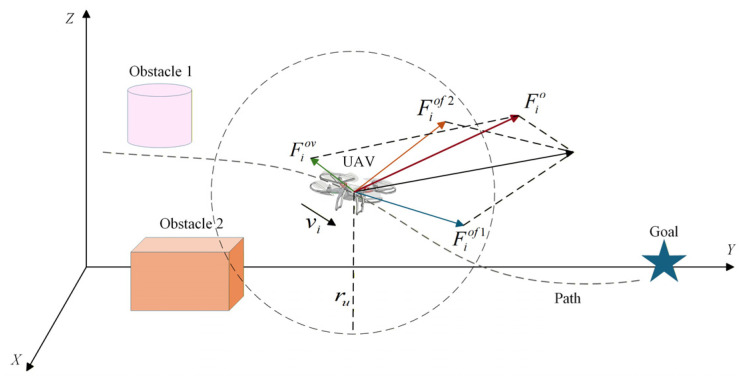
Schematic diagram of obstacle-avoidance control force.

The repulsive force analysis of UAV i under the obstacle-avoidance control law is illustrated in Figure 9.

#### 3.2.3. Formation Cooperative Control Law

To ensure stable tracking and formation consistency in complex environments, a distributed cooperative controller based on a virtual leader is designed. By integrating position errors, velocity errors, and communication topology information, the proposed method enhances formation consistency maintenance and formation recovery during obstacle-avoidance maneuvers.

The virtual leader is the core of global path tracking, with position $p_{0}$ and velocity $v_{0}$. The desired position of UAV i is defined as $p_{i}^{d}=p_{0}-d_{i}$, where $d_{i}=[d_{i}^{x},d_{i}^{y},d_{i}^{z}{]}^{T}$ denotes the position offset of the i-th UAV relative to the leader. The position and velocity tracking errors of UAV i are defined as:

(39) $$\left\{\begin{array}{c} e_{i}^{p}=p_{i}-p_{i}^{d}\\ e_{i}^{v}=v_{i}-v_{i}^{d} \end{array}\right.$$

Based on the distributed architecture and multi-agent consensus theory, the formation control input $F_{i}^{f}$ is designed as:

(40) $$F_{i}^{f}=-k_{1}^{f}\sum_{j=1}^{n}a_{ij}\left(e_{i}^{p}-e_{j}^{p}\right)-k_{2}^{f}\sum_{j=1}^{n}a_{ij}\left(e_{i}^{v}-e_{j}^{v}\right)-k_{1}^{f}a_{i0}e_{i}^{p}-k_{2}^{f}a_{i0}e_{i}^{v}$$

where $a_{ij}$ is an element of the adjacency matrix, indicating the communication link between UAVs i and j ($a_{ij}=1$ if connected, otherwise 0); $a_{i0}$ denotes the connection status between UAV i and the virtual leader; $k_{1}^{f}>0$ is the position error feedback gain, which primarily adjusts the convergence speed of the formation position; $k_{2}^{f}>0$ is the velocity error feedback gain, which mainly adjusts velocity consistency and damping characteristics, used to suppress oscillations and improve convergence stability.

**Figure 10:**
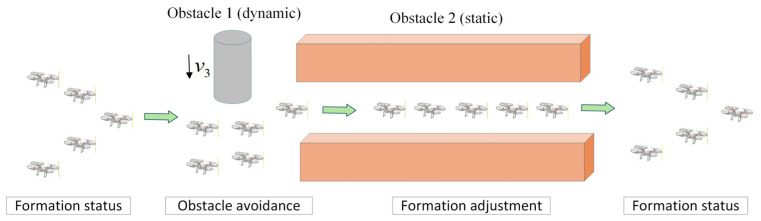
Schematic of formation-state transitions under the coordinated action of the control laws.

Figure 10 illustrates the evolution of the UAV formation state under the coordinated action of the three control laws. In open areas, the cooperative formation controller ensures stable formation maintenance. When dynamic obstacles appear, the external obstacle-avoidance control law dominates trajectory adjustment to achieve safe avoidance. Upon entering a narrow passage, the internal collision-avoidance control and the cooperative formation control act jointly, driving the formation to transition from its original configuration to a compact shape suitable for passage. After clearing the obstacle region, the formation returns to a stable state.

### 3.3. System Cooperative Mechanism

#### 3.3.1. Virtual Leader

In conventional leader–follower architectures, maneuvering errors or failures of a physical leader propagate to followers, posing a threat to overall formation stability. To mitigate this single-point-of-failure risk, a virtual leader–follower strategy is adopted. The virtual leader generates the formation trajectory in real time based on environmental information and the path produced by the DASRRT* algorithm (Section 3.1), while follower UAVs track its motion via the cooperative formation control law (Section 3.2.3). Driven by multi-objective control inputs, the virtual leader dynamics are formulated as follows:

(41) $$\left\{\begin{array}{c} \dot{p_{0}}=v_{0}\\ \dot{v_{0}}=-\gamma_{1}v_{0}+F_{virtual}=-\gamma_{1}v_{0}+F_{att}+F_{rep}+F_{form} \end{array}\right.$$

(42) $$F_{att}=k_{att}\left(s_{local}-p_{0}\right)$$

(43) $$F_{form}=k_{form}\left(\frac{1}{N}\sum_{i=1}^{N}p_{i}-p_{0}\right)$$

where $\gamma_{1}$ denotes the velocity damping factor used to suppress oscillations, $F_{att}$ represents the target attraction force, $s_{local}$ denotes a local target point dynamically selected along the global path in real-time (Section 3.3.2), $k_{att}$ denotes the attractive gain coefficient, $F_{rep}$ denotes the obstacle repulsive force, and $F_{form}$ denotes the formation center regression force. The dynamic gain coefficient $k_{form}$ is computed as follows:

(44) $$k_{form}=\left\{\begin{array}{c} k_{form}^{0}e^{-\gamma_{2}\frac{1}{\left\|p_{0}-p_{goal}\right\|}},\;\;\;\left\|p_{0}-p_{goal}\right\|>\delta_{goal}\\ 0,\;\;\;\mathrm{otherwise} \end{array}\right.$$

where $k_{form}^{0}$ denotes the baseline regression gain, $\gamma_{2}$ denotes the attenuation factor, and $\delta_{goal}$ denotes the target distance threshold. As the leader approaches the final destination, formation constraints are gradually relaxed to avoid target inaccessibility.

**Figure 11:**
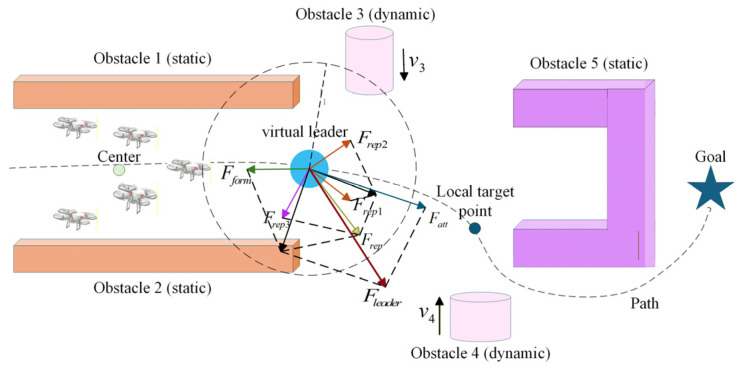
Force analysis of the virtual leader.

The forces acting on the virtual leader are illustrated in Figure 11.

#### 3.3.2. Local Target Selection Strategy

To address the limited adaptability of fixed-step local target switching in complex environments, a curvature-driven dynamic step-length strategy is proposed to smooth formation speed, mitigate oscillations caused by a constant step size, and improve overall formation stability.

(1) Curvature-driven step-length adjustment

When the virtual leader tracks the global path, the local target point $p_{local}$ is adjusted dynamically according to the local path curvature k. An adaptive step length Δs is designed to select $p_{local}$ in real time.

(45) $$∆s=∆s_{min}+\left(∆s_{max}-∆s_{min}\right)\frac{1}{1+e^{\alpha \left(k\left(s\right)-k\right)}}$$

where $\Delta s_{min}$ is the minimum step length, $\Delta s_{max}$ is the maximum step length, α is the curvature coefficient, and k is the curvature threshold. k(path) denotes the path curvature, which characterizes the degree of bending; s is the arc length. Larger k(s) indicates sharper turns, while k(s)→0  implies the path approaches a straight line. In high-curvature regions $\left(k(s)\geq k_{th}\right)$, denser sampling is required to improve obstacle-avoidance accuracy and ensure dense waypoints at sharp turns to prevent collisions; in low-curvature regions $\left(k(s)<k_{th}\right)$, sampling can be sparser to improve efficiency and accelerate tracking along straight segments.

(2) Local Target Selection

For path tracking, the virtual leader moves along the optimized global path and dynamically selects the local target point $p_{local}$. The procedure is as follows.

First, compute the Euclidean distance from the virtual leader’s current position $p_{0}$ to the path π and determine the nearest path point:

(46) $$p_{nearest}=arg\underset{k}{\min}{\left\|p_{0}-p_{k}\right\|}_{2}$$

Then, starting from $p_{nearest}$, move forward along the path by the adaptive step length Δs:

(47) $$p_{target}=p_{nearest}+\Delta s$$

where $p_{target}$ is the local target point. When the distance between $p_{nearest}$ and the goal is less than $\Delta s(p_{nearest})$, the next local target is the goal.

#### 3.3.3. Dual-Mode Switching Mechanism

To meet formation control requirements in complex terrain, a dual-mode switching mechanism is proposed, and mode switching is triggered by the environmental complexity η. The environmental complexity η is defined as the obstacle density within a local region:

(48) $$\eta =\frac{1}{N_{obs}}\sum_{o\in O_{i}}\frac{1}{\left\|p_{i}-p_{o}\right\|+\varepsilon }$$

where $N_{obs}$ is the number of obstacles within the sensing range, ε is a small positive constant to avoid a zero denominator, and $O_{i}$ is the set of obstacles within the sensing radius $r_{u}$ of UAV i, i.e., $O_{i}=\{o|\left\|p_{i}-p_{o}\right\|\leq R_{sense}\}$. A larger η indicates a more complex environment, meaning obstacles are denser around the UAV (e.g., narrow corridors or obstacle clusters).

The switching logic is defined as:

(49) $$Mode=\left\{\begin{array}{c} \text{formation}-\text{maintenance }\;\text{mode},\;\;\eta \leq \eta_{1\;}\text{and}\;\left\|e_{i}^{p}\right\|\leq \delta_{safe}\\ \text{emergency}-\text{avoidance }\;\text{mode},\;\;\eta >\eta_{1\;}\text{or}\;\left\|e_{i}^{p}\right\|>\delta_{safe}\;\; \end{array}\right.$$

where $\eta_{1}$ is the environmental complexity threshold; $\left\|e_{i}^{p}\right\|=\left\|p_{i}-(p_{0}+d_{i})\right\|$ is the position error between the actual and desired locations; $\delta_{safe}$ is the safety threshold for formation distance.

##### Formation Maintenance Mode (Mode 1)

The total control input of each UAV, $F_{i}(t)$, is a weighted sum of the formation control force, the internal collision-avoidance force, and the external obstacle-avoidance force:

(50) $$F_{i}\left(t\right)=k_{u}^{1}F_{i}^{f}\left(t\right)+k_{u}^{2}\sum_{j=1,j\neq i}^{N}F_{ji}^{c}\left(t\right)+k_{u}^{3}\sum_{o=1}^{K}F_{io}^{o}\left(t\right)$$

where $F_{ji}^{c}$ is the collision-avoidance control force between UAV i and its neighbor j; $F_{io}^{o}$ is the obstacle-avoidance control force on UAV i from obstacle o; $F_{i}^{f}$ is the formation consensus control force. $k_{u}^{1}$, $k_{u}^{2}$, and $k_{u}^{3}$ are weighting coefficients that balance the priorities between formation maintenance and collision/obstacle avoidance.

$k_{u}^{1}$ is the weight of formation control. Its primary role is to enforce formation maintenance; in open environments it strengthens formation consensus to ensure accurate tracking of the desired shape led by the virtual leader. In narrow or obstacle-dense regions, reducing $k_{u}^{1}$ weakens formation constraints and enhances collision/obstacle avoidance. Therefore, $k_{u}^{1}$ is designed as an inverse proportional saturation function that adapts to the environmental complexity η:

(51) $$k_{u}^{1}=max\left(k_{1}^{max}\left(1-\frac{\eta }{\eta_{1}}\right),k_{1}^{min}\right)$$

where $k_{1}^{max}$ and $k_{1}^{min}$ are the maximum and minimum weights of the formation control force, and $\eta_{1}$ is the environmental complexity threshold. As η→0, $k_{u}^{1}\to k_{1}^{max}$, so formation control dominates; as $\eta \to \eta_{1}$, $1-\eta /\eta_{1}\to 0$ and $k_{u}^{1}\to k_{1}^{min}$, thereby reducing the formation control strength.

$k_{u}^{2}$ and $k_{u}^{3}$ are the weights for collision avoidance and obstacle avoidance, respectively, and are designed with linear and exponential variations:

(52) $$k_{u}^{2}=k_{2}^{0}\left(1+\frac{\eta }{\eta_{1}}\right)$$

(53) $$k_{u}^{3}=k_{3}^{0}\left(\frac{\eta -\eta_{1}}{\sigma }\right)$$

where $k_{u}^{2}$ increases linearly to enhance inter-UAV collision avoidance, and $k_{u}^{3}$ increases exponentially to strengthen obstacle repulsion. The parameter σ controls the growth rate of the gain. For small σ(e.g., σ=0.3), $k_{u}^{3}$ rises sharply once $\eta_{1}$ is exceeded, which suits highly dynamic obstacle scenarios; for larger σ(e.g., σ=1.0), the gain varies smoothly, which is suitable for environments with uniformly distributed obstacles.

**Figure 12:**
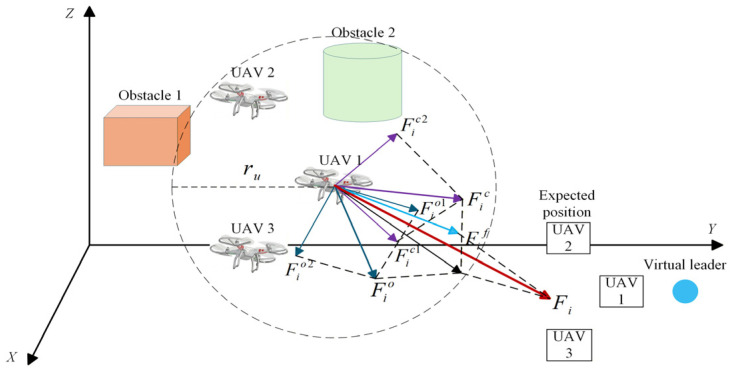
Force analysis in formation mode.

Considering variations in environmental complexity across scenarios, the dynamic adjustment of $k_{u}^{1}$, $k_{u}^{2}$, and $k_{u}^{3}$ balances safety and formation efficiency. Formation maintenance is prioritized in open environments, while collision and obstacle avoidance are prioritized in confined environments. The total control force of UAV i is shown in Figure 12.

##### Emergency-Avoidance Mode (Mode 2)

Using only the control law of formation maintenance mode may be insufficient in extreme, highly complex environments. Therefore, an emergency-avoidance mode is introduced to strengthen the goal-attractive and obstacle-repulsive forces. The control input is adjusted as:

(54) $$F_{i}\left(t\right)=k_{u}^{1}F_{i}^{att}\left(t\right)+k_{u}^{2}\sum_{j=1,j\neq i}^{N}F_{ji}^{c}\left(t\right)+k_{u}^{3}\sum_{o=1}^{K}F_{io}^{o}\left(t\right)$$

In this mode, the attractive force $F_{i}^{att}$ consists of two components:

(55) $$F_{i}^{att}=F_{i}^{att1}+F_{i}^{att2}=k_{att}^{1}\left(p_{0}-p_{i}\right)+k_{att}^{2}\left(p_{nearest}-p_{i}\right)$$

where $F_{i}^{att1}$ is the attraction toward the virtual leader, driving the UAV to follow the leader’s position $p_{0}$ and maintain formation coherence; $F_{i}^{att2}$ is the attraction toward the local path point, providing global-path guidance by steering the UAV toward the nearest reachable path point $p_{nearest}$, thereby enhancing path-tracking robustness. $k_{attr}^{1}$ and $k_{attr}^{2}$ are the attraction gains.

In high-curvature regions, path points are dense and $p_{nearest}$ updates frequently, so the attraction direction adapts quickly to the path; in low-curvature regions, points are sparse and the attraction direction is more stable, reducing control oscillations. As the core driving force in the emergency-avoidance mode, $F_{i}^{att}$ combines attraction to the virtual leader and the local path to ensure formation coherence and real-time avoidance in complex terrain.

The proposed dual-mode switching mechanism temporarily switches to the emergency-avoidance mode in particularly complex environments, where $F_{i}^{att}$ dominates the cooperative control input to guide the UAVs to rapidly and effectively exit obstacle regions under the combined guidance of the virtual leader and the global path; otherwise, the formation maintenance mode is used.

## 4. Stability and Convergence Analysis

This chapter conducts a rigorous stability analysis of the control law proposed in Section 3.

**Lemma 1** (LaSalle Invariance Principle)**.**  *For a differentiable function* V *defined on* $R^{n}$*, if* V(x)≥0 *and* V(x)=0 *if and only if* x=0*, and if* $\dot{V}(x)\leq 0$ *for all* $x\in R^{n}$*, then as* t→∞ *the system trajectory* x(t) *converges to the set* $S=\{\dot{V}(x)=0\}$*. If* S *contains no invariant set other than* $\left\{0\right\}$*, then the trajectory* x(t) *is asymptotically stable [40]*.

### 4.1. Stability Analysis of the Collision-Avoidance Control Law

The collision-avoidance control law accounts for the relative positions and relative velocities among UAVs, ensuring that no collisions occur between any two UAVs. To rigorously establish the stability of the proposed control law and verify that the safety-distance constraint is always satisfied, we construct the following Lyapunov function:

(56) $$V_{c}\left(t\right)=\frac{1}{2}\sum_{i=1}^{N}\sum_{j=1,j\neq i}^{N}U_{c}\left(\left\|p_{ji}\right\|\right)+\frac{1}{2}\sum_{i=1}^{N}v_{i}^{T}v_{i}$$

This Lyapunov function consists of a potential-energy term and a kinetic-energy term. Taking the time derivative yields

(57) $$\begin{array}{ll} \dot{V_{c}}\left(t\right)=\frac{1}{2}\sum_{i=1}^{N}\sum_{j=1,j\neq i}^{N} & \left(\frac{dU_{c}}{d\left\|p_{ji}\right\|}\frac{d\left\|p_{ji}\right\|}{dt}\right)+\frac{1}{2}\sum_{i=1}^{N}\left({\dot{v_{i}}}^{T}v_{i}+{v_{i}}^{T}\dot{v_{i}}\right)\\ & =\frac{1}{2}\sum_{i=1}^{N}\sum_{j=1,j\neq i}^{N}\left(-\frac{k_{1}^{c}}{{\left(\left\|p_{ji}\right\|-r_{s}\right)}^{3}}\frac{{p_{ji}}^{T}\dot{p_{ji}}}{\left\|p_{ji}\right\|}\right)+\sum_{i=1}^{N}{v_{i}}^{T}\dot{v_{i}}\\ & =\frac{1}{2}\sum_{i=1}^{N}\sum_{j=1,j\neq i}^{N}\left(-\frac{k_{1}^{c}}{{\left(\left\|p_{ji}\right\|-r_{s}\right)}^{3}}\frac{{p_{ji}}^{T}\dot{p_{ji}}}{\left\|p_{ji}\right\|}\right)\\ & -\sum_{i=1}^{N}\sum_{j=1,j\neq i}^{N}\left(v_{i}^{T}\frac{k_{1}^{c}}{{\left(\left\|p_{ji}\right\|-r_{s}\right)}^{3}}\frac{p_{ji}}{\left\|p_{ji}\right\|}\right)-\sum_{i=1}^{N}\sum_{j=1,j\neq i}^{N}{v_{i}}^{T}k_{2}^{c}v_{ji} \end{array}$$

For the second term on the right-hand side of the last equality in (57), we analyze the subsystem composed of UAV i and UAV j. For a pair $\left(i,j\right)$ without an edge, when i is included in the outer summation and j in the inner summation, the contribution to the subsystem total energy is

(58) $${v_{i}}^{T}\left(-\frac{k_{1}^{c}}{{\left(\left\|p_{ji}\right\|-r_{s}\right)}^{3}}\frac{{p_{ji}}^{T}}{\left\|p_{ji}\right\|}\right)$$

When j is included in the outer summation and i in the inner summation, the contribution to the subsystem total energy is

(59) $${v_{j}}^{T}\left(-\frac{k_{1}^{c}}{{\left(\left\|p_{ij}\right\|-r_{s}\right)}^{3}}\frac{{p_{ij}}^{T}}{\left\|p_{ij}\right\|}\right)={v_{j}}^{T}\left(-\frac{k_{1}^{c}}{{\left(\left\|p_{ji}\right\|-r_{s}\right)}^{3}}\frac{{p_{ji}}^{T}}{\left\|p_{ji}\right\|}\right)$$

Since the forces acting on the two UAVs have equal magnitude and opposite direction, we have:

(60) $${v_{i}}^{T}\left(-\frac{k_{1}^{c}}{{\left(\left\|p_{ji}\right\|-r_{s}\right)}^{3}}\frac{{p_{ji}}^{T}}{\left\|p_{ji}\right\|}\right)=-\frac{1}{2}\left({v_{i}}^{T}+{v_{j}}^{T}\right)\frac{k_{1}^{c}}{{\left(\left\|p_{ji}\right\|-r_{s}\right)}^{3}}\frac{{p_{ji}}^{T}}{\left\|p_{ji}\right\|}\;=-\frac{1}{2}\frac{k_{1}^{c}}{{\left(\left\|p_{ji}\right\|-r_{s}\right)}^{3}}\frac{{p_{ji}}^{T}}{\left\|p_{ji}\right\|}v_{ji}$$

Substituting (60) into (57) yields

(61) $$\dot{V_{c}}\left(t\right)=-\sum_{i=1}^{N}\sum_{j=1,j\neq i}^{N}\left({v_{i}}^{T}k_{2}^{c}v_{ji}\right)=-\sum_{\left(i,j\right)\in E}k_{2}^{c}\left[{v_{i}}^{T}\left(v_{j}-v_{i}\right)+{v_{j}}^{T}\left(v_{i}-v_{j}\right)\right]\;\;\;\;\;\;\;=-\sum_{\left(i,j\right)\in E}k_{2}^{c}{\left(v_{j}-v_{i}\right)}^{T}\left(v_{j}-v_{i}\right)=-\sum_{\left(i,j\right)\in E}k_{2}^{c}{\left\|v_{ji}\right\|}^{2}\leq 0$$

From (61), it follows that ${\dot{V}}_{c}(t)\leq 0$, and from (56), $V_{c}(t)>0$. According to LaSalle’s invariance principle, the system asymptotically converges to the largest invariant set satisfying ${\dot{V}}_{c}(t)=0$, and $V_{c}(t)\leq V_{c}(0)$. When $∥p_{ji}∥\to r_{s}$, $V_{c}(t)\to \infty$, which contradicts the boundedness established above. Therefore, if the initial inter-UAV distances exceed the prescribed safety distance, collisions will not occur.

### 4.2. Stability Analysis of the Obstacle-Avoidance Control Law

To demonstrate that the UAV can safely avoid obstacles under the obstacle-avoidance control law and ultimately converge to the target point, the following Lyapunov function is constructed:

(62) $$V_{o,i}\left(t\right)=U_{i}^{o}\left(p_{i}\right)+\frac{1}{2}{v_{i}}^{T}v_{i}$$

The first term represents the obstacle-repulsive potential energy, while the second term corresponds to the kinetic energy. Taking the time derivative gives

(63) $${\dot{V}}_{o,i}(t)=U_{i}^{o}\left(p_{i}\right)+\frac{1}{2}{v_{i}}^{T}v_{i}=\nabla_{p_{i}}U_{i}^{o}v_{i}+{v_{i}}^{T}\left(-\nabla_{p_{i}}U_{i}^{o}-k_{4}^{o}v_{i}\right)=-k_{4}^{o}{\left\|v_{i}\right\|}^{2}\leq 0$$

From (63), the system energy is non-increasing, i.e., ${\dot{V}}_{o,i}(t)\leq 0$, and $V_{o,i}(t)>0$. Consequently, the system asymptotically converges to the largest invariant set satisfying ${\dot{V}}_{o,i}(t)=0$, with $V_{o,i}(t)\leq V_{o,i}(0)$. Hence, the potential energy term remains bounded. When the distance between the UAV and the obstacle approaches zero, $V_{o,i}(t)\to \infty$, which contradicts the boundedness established above. It follows that, during formation flight, the obstacle-avoidance control law guarantees that the UAV does not collide with obstacles.

### 4.3. Stability Analysis of Formation Errors Under the Control Law

In actual formation flight, UAVs are not solely influenced by a single type of control force, but instead move under the combined influence of formation coordination control forces, inter-UAV collision-avoidance forces, and external obstacle-avoidance forces. Therefore, this section introduces collision-avoidance and obstacle-avoidance forces as active evasion inputs into the formation error system, and combines them with the formation coordination control law. The boundedness and convergence recovery of the errors under the overall control law are analyzed from the perspective of the overall closed-loop system.

According to Equation (50), the total control force on the i-th UAV during formation flight is:

(64) $$F_{i}=k_{u}^{1}F_{i}^{f}+k_{u}^{2}\sum_{j=1,j\neq i}^{N}F_{ji}^{c}+k_{u}^{3}\sum_{o=1}^{K}F_{io}^{o}$$

The avoidance inputs are expressed as:

(65) $$d_{i}=k_{u}^{2}\sum_{j=1,j\neq i}^{N}F_{ji}^{c}+k_{u}^{3}\sum_{o=1}^{K}F_{io}^{o}$$

Define position error as $e^{p}=[(e_{1}^{p}{)}^{T},(e_{2}^{p}{)}^{T},\ldots ,(e_{N}^{p}{)}^{T}]$, and velocity error as $e^{v}=[(e_{1}^{v}{)}^{T},(e_{2}^{v}{)}^{T},\ldots ,(e_{N}^{v}{)}^{T}]$. The main avoidance input is $d=[d_{1}^{T},d_{2}^{T},\ldots ,d_{N}^{T}]$. Combining the concepts in Section 2, the formation error system is written as:

(66) $$\left\{\begin{array}{c} \dot{e^{p}}=e^{v}\;\\ \dot{e^{v}}=-k_{1}^{f}\left(\left(L+H\right)⨂I_{3}\right)e^{P}-k_{2}^{f}\left(\left(L+H\right)⨂I_{3}\right)e^{v}+d \end{array}\right.$$

where L is the Laplacian matrix of the communication topology, and H represents the connection matrix between the leader and followers. The symbol ⊗ indicates the Kronecker product, and $I_{3}$ is the 3D identity matrix. When the UAVs are in communication and can efficiently receive the leader’s information, L+H is positive definite, i.e., $\lambda_{\min}(L+H)>0$.

First, analyze the avoidance input when d=0, so the formation error system can be written as:

(67) $$\left\{\begin{array}{c} \dot{e^{p}}=e^{v}\\ \dot{e^{v}}=-k_{1}^{f}\left(\left(L+H\right)⨂I_{3}\right)e^{P}-k_{2}^{f}\left(\left(L+H\right)⨂I_{3}\right)e^{v} \end{array}\right.$$

For the Lyapunov function corresponding to Equation (67), we construct:

(68) $$V_{f}=\frac{1}{2}k_{1}^{f}{\left(e^{p}\right)}^{T}\left(\left(L+H\right)⨂I_{3}\right)e^{p}+\frac{1}{2}{\left(e^{v}\right)}^{T}e^{v}$$

Since L+H is positive definite and $k_{1}^{f}>0$, we have:

(69) $$V_{f}>0$$

From Equation (68), we get:

(70) $$\begin{array}{c} \dot{V_{f}}=k_{1}^{f}{\left(e^{p}\right)}^{T}\left(\left(L+H\right)⨂I_{3}\right)\dot{e^{p}}+{\left(e^{v}\right)}^{T}\dot{e^{v}}\\ =k_{1}^{f}{\left(e^{p}\right)}^{T}\left(\left(L+H\right)⨂I_{3}\right)e^{v}+{\left(e^{v}\right)}^{T}\left[-k_{1}^{f}\left(\left(L+H\right)⨂I_{3}\right)e^{P}-k_{2}^{f}\left(\left(L+H\right)⨂I_{3}\right)e^{v}\right]\\ =-k_{2}^{f}\left(\left(L+H\right)⨂I_{3}\right)e^{v}\leq 0 \end{array}$$

As seen from (70), $V_{f}$ is not an increment. According to LaSalle’s invariance principle, the system’s trajectory will eventually converge to the largest invariant set where $V_{f}=0$. When $e^{v}=0$, we substitute into Equation (67) to get $k_{1}^{f}\left((L+H)⊗I_{3}\right)e^{P}=0$. Since $k_{1}^{f}>0$, and $(L+H)⊗I_{3}$ is positive definite, it follows that $e^{p}=0$. Therefore, under the condition of zero avoidance input, the formation control law’s stability can be ensured:

(71) $$\underset{t\to \infty }{\lim}{\;e}^{p}\left(t\right)=0,\;\;\;\underset{t\to \infty }{\lim}e^{v}\left(t\right)=0$$

Next, we analyze the case with non-zero avoidance input, d≠0. In this case, from Equation (66), the formation error system can be written as:

(72) $$\dot{V_{f}}=-k_{2}^{f}\left(\left(L+H\right)⨂I_{3}\right)e^{v}+{\left(e^{v}\right)}^{T}d$$

From Equation (64), when active collision-avoidance forces and obstacle avoidance forces are present, the term $\left(e^{v}{)}^{T}d\right.$ acts as an external input to the error system. Under these conditions, it cannot be deduced that ${\dot{V}}_{f}\leq 0$, nor can it be directly proven that the tracking error strictly asymptotically converges to zero throughout the entire obstacle-avoidance process. In fact, during the obstacle-avoidance phase, the UAVs need to temporarily deviate from the desired formation to satisfy the constraints on inter-UAV safety distance and obstacle safety distance. Therefore, the occurrence of short-term fluctuations in the tracking error is reasonable.

To further demonstrate the role of avoidance input in affecting the error dynamics, Equation (66) is rewritten in state-space form as:

(73) $$\dot{\xi }=A_{f}\xi +B_{f}d$$

This leads to:

(74) $$\left(\begin{array}{c} \dot{e^{p}}\\ \dot{e^{v}} \end{array}\right)=\left(\begin{array}{cc} 0 & I_{3N}\\ -k_{1}^{f}\left(L+H\right)⨂I_{3} & -k_{2}^{f}\left(L+H\right)⨂I_{3} \end{array}\right)\left(\begin{array}{c} e^{p}\\ e^{v} \end{array}\right)+\left(\begin{array}{c} 0\\ I_{3N} \end{array}\right)d$$

From the analysis of the error system above, it is known that when L+H is positive definite and $k_{1}^{f}>0,k_{2}^{f}>0$, the matrix $A_{f}$ is a Hurwitz matrix. Therefore, there exist positive constants c>0  and α>0 such that:

(75) $$\left\|e^{A_{f}t}\right\|\leq ce^{-\alpha t}$$

If the avoidance input is bounded, i.e., there exists a positive constant $\bar{d}$ satisfying:

(76) $$\left\|d\left(t\right)\right\|\leq \bar{d}$$

Then Equation (73) yields:

(77) $$\xi \left(t\right)=e^{A_{f}t}\xi \left(0\right)+\int_{0}^{t}e^{A_{f}\left(t-\tau \right)}\left\|B_{f}\right\|\left\|d\left(\tau \right)\right\|d\tau$$

Then we have:

(78) $$\left\|\xi \left(t\right)\right\|\leq ce^{-\alpha t}\left\|\xi \left(0\right)\right\|+\int_{0}^{t}e^{A_{f}\left(t-\tau \right)}\left\|B_{f}\right\|\left\|d\left(\tau \right)\right\|d\tau \leq ce^{-\alpha t}\left\|\xi \left(0\right)\right\|+\frac{c\left\|B_{f}\right\|}{\alpha }\bar{d}$$

From Equation (78), it can be seen that the formation tracking error will not diverge, but remains within a bounded neighborhood related to $\overset{ˉ}{d}$.

In summary, the overall control law designed exhibits the following stability characteristics in the closed-loop system: in the absence of evasion inputs, the formation error asymptotically converges to zero; when evasion inputs are present, the control force—as an external input—causes the UAVs to temporarily deviate from the desired formation, but the formation error remains bounded; once the UAVs move away from the obstacle, the formation tracking error recovers and asymptotically converges to zero. This conclusion aligns with actual flight behavior: when the formation enters an area with complex obstacles, it cannot fully maintain its original formation and must prioritize obstacle avoidance; the formation tracking error may temporarily increase but remains within a controlled range; after leaving the obstacle area, the formation is able to recover and maintain its original formation.

## 5. Simulation Experiments

**Figure 13:**
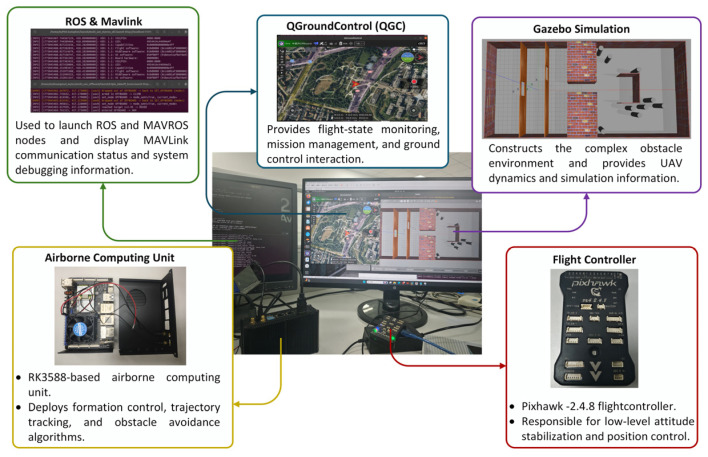
Gazebo-based hardware-in-the-loop simulation platform.

The simulations were conducted on a laptop running the Windows 11 operating system (Intel(R) Core(TM) i7-10750H processor). All programs were implemented in MATLAB R2022b. In addition, to further verify the feasibility and closed-loop operational capability of the proposed method under conditions closer to practical system deployment, a Gazebo-based hardware-in-the-loop simulation platform was established, as shown in Figure 13. The platform consists of a host computer, a Pixhawk 2.4.8 flight controller, an RK3588-based onboard computing unit, the QGroundControl (QGC) ground control station, and the Gazebo simulation environment. Specifically, the host computer is responsible for running the Gazebo scenario, ROS/MAVROS communication nodes, and the state-monitoring interface; the RK3588 onboard computing unit is used to deploy the formation control, trajectory tracking, and obstacle-avoidance algorithms; the Pixhawk flight controller handles low-level attitude stabilization and position control; and QGC is used for flight-state monitoring and mission management. Closed-loop interaction of state information, control commands, and simulation data among the modules is achieved through MAVLink and ROS communication, thereby forming an integrated verification chain of “simulation environment–onboard computing–flight control–ground monitoring.”

Considering the characteristics of low-altitude combat environments, a three-dimensional integrated simulation scenario containing multiple heterogeneous obstacles was constructed. The scene replicates a typical complex terrain characterized by constrained spatial structures and dynamic threats. The static environment includes U-shaped obstacles, narrow passages, walls with windows, and clusters of tree-like obstacles. In addition, randomly moving cylindrical dynamic obstacles are introduced to emulate unknown threats.

### 5.1. Simulation Validation of the Path Optimization Algorithm

#### 5.1.1. Comparative Experiments

In this section, the proposed DASRRT* algorithm is compared with the RRT* algorithm, the Informed RRT* algorithm [41], the RRT*-APF algorithm [42], the RRT*-PRIME algorithm [43], and the BIT* algorithm. The simulation is conducted in a three-dimensional space of 150 m×60 m×20 m. The leader UAV starts from $\left(2\;m,30\;m,5\;m\right)$ and the target position is set to $\left(145m,30m,5m\right)$. To ensure the fairness and reproducibility of the comparison experiments, the common parameters for the RRT* series algorithms are given as follows: maximum number of iterations $N_{\max}=7000$, base extension step size $\delta_{\max}=2\;m$, neighborhood rewire radius $r_{\mathrm{rewire}}=10\;m$, goal arrival threshold $\epsilon_{\mathrm{goal}}=3\;m$, and obstacle safety margin $\delta_{\mathrm{safe}}=0.8\;m$. In addition, the specific parameters for each comparison algorithm are listed in Table 1.

In Table 1, $P_{g}$ is the target bias sampling probability; $\gamma_{IRRT*}$ is the dynamic neighborhood radius adjustment coefficient for Informed RRT*; $k_{\mathrm{APF}}$, $r_{u}$, and $\alpha_{\mathrm{APF}}$ are the APF repulsive gain, obstacle influence radius, and APF sampling point correction step size in RRT-APF*, respectively. $w_{1}$, $w_{2}$, and $w_{3}$ are the weights for distance, direction consistency, and obstacle distribution; $P_{\mathrm{tar}}$ is the heuristic probability threshold; $K_{\mathrm{cand}}$ is the number of candidate directions; and $\theta_{\mathrm{bias}}$ is the angular bias range. $w_{4}$, $w_{5}$, and $w_{6}$ are the weights for path length, obstacle safety, and smoothness; $C_{\mathrm{th}}$ and $E_{\mathrm{th}}$ are the cost growth threshold and emergency adjustment threshold, respectively; $\gamma_{1}$, $\gamma_{2}$, and κare used for obstacle density estimation. In BIT*, $N_{s}$ is the maximum number of sampling points, $N_{b}$ is the batch size for sampling, $\eta_{\mathrm{BIT}}$ is the connection radius adjustment coefficient, and $r_{\min}$ and $r_{\max}$ are the minimum and maximum connection radii, respectively. In DASRRT*, $P_{f}$ is the hybrid guided sampling probability, ρ and λ are the scaling factor and shape parameter in dynamic focus sampling, β is the perceptual adaptive step size sensitivity parameter, $\Delta s_{\mathrm{smooth}}$ is the path smoothing sampling interval, and $\delta_{\min}$ is the adaptive step size lower limit.

Regarding the termination conditions, RRT*, Informed RRT*, RRT-APF*, RRT-PRIME*, and DASRRT* all stop after reaching the maximum iteration count of $N_{\max}=7000$. For the BIT* algorithm, due to its use of batch sampling and graph search mechanism, which differs from the point-by-point sampling strategy of the RRT* series algorithms, the termination condition is set to a cumulative number of sampling points reaching 7000 to align the total sampling scale with that of the RRT* series algorithms.

**Figure 14:**
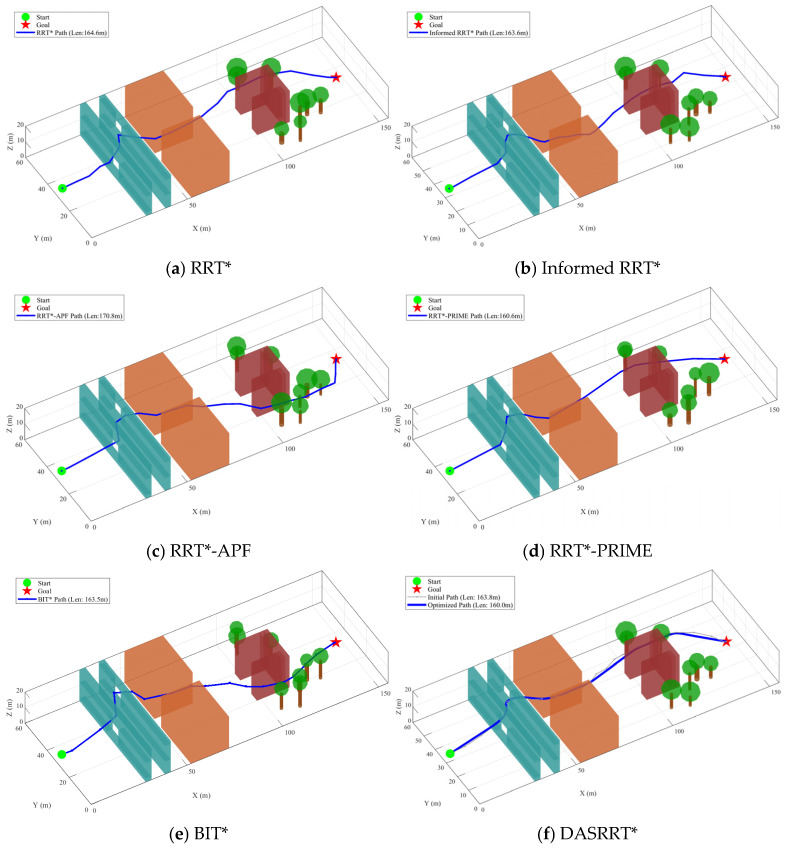
Planning results of the six algorithms.

As shown in Figure 14, although all comparative algorithms are able to find feasible paths in a complex three-dimensional environment, the resulting paths exhibit pronounced piecewise polyline characteristics. Owing to the large turning-angle discontinuities along these paths, UAVs are often required to perform frequent aggressive acceleration, deceleration, and large attitude adjustments during tracking, which not only increases control difficulty but also leads to higher energy consumption. In contrast, the path generated by DASRRT* is smoother. By introducing a path optimization strategy, the proposed algorithm effectively eliminates abrupt turns while maintaining a relatively short path length, making it more suitable as a reference trajectory for UAV formation tracking control.

**Figure 15:**
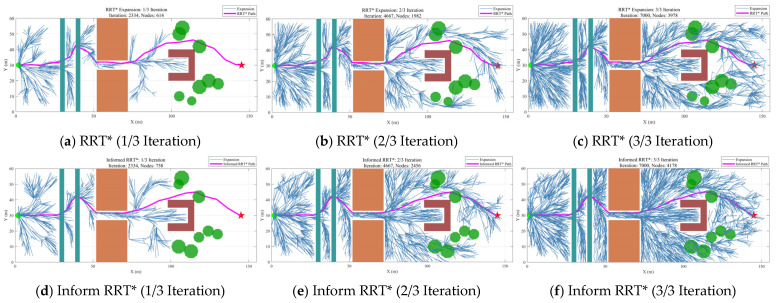

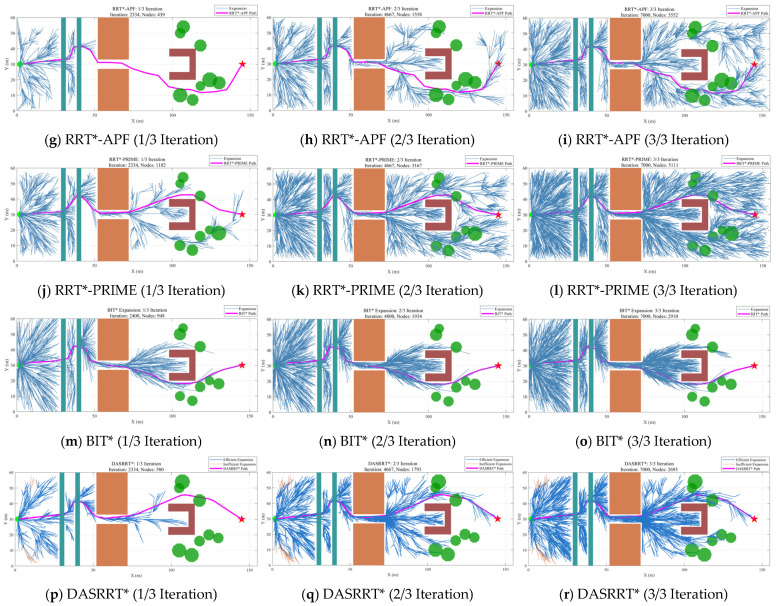
Comparison of the expansion-tree evolution of the algorithms.

To further reveal the fundamental differences among the algorithms in spatial search behavior, Figure 15 presents the evolution of the expansion trees at 1/3, 2/3, and 3/3 of the total iterations. Although Informed RRT* reduces the search region through elliptical heuristic sampling, its expansion tree still exhibits a relatively high node density. RRT*-APF shows a certain degree of directionality under potential-field guidance, but its overall expansion process remains highly stochastic. Although RRT*-PRIME has an advantage in path length, its expansion tree becomes extremely dense at the final iteration stage, indicating that a substantial amount of computational resources is consumed by repeated exploration in non-optimal regions. In contrast, the expanded trees of BIT* and DASRRT* exhibit a more pronounced goal-oriented nature, with DASRRT* demonstrating superior performance in terms of path length and smoothness. Consequently, DASRRT* not only significantly reduces tree size and computational overhead but also generates smooth, high-quality paths while maintaining high search efficiency.

Given the inherent randomness of sampling-based algorithms, results from a single trial are insufficient for an objective assessment of robustness. Therefore, a Monte Carlo simulation was conducted, where each algorithm was independently repeated 50 times under the same environment. For each performance metric, the mean, standard deviation, and 95% confidence interval were calculated, and a *t*-test was performed between DASRRT* and each comparison algorithm. A difference was considered statistically significant when p<0.05. The statistical results are shown in Table 2 and Table 3.

As shown in Table 2 and Table 3, the average path length of DASRRT* is 161.13 m, which is reduced by 3.54%, 2.66%, 1.84%, and 1.36% compared with RRT*, Informed RRT*, RRT*-APF, and BIT*, respectively. The path cost differences between DASRRT* and RRT*, Informed RRT*, and RRT*-APF are statistically significant. While RRT*-PRIME has the shortest average path, its node count and solving time are 5276.53 and 49.83 s, which are significantly higher than those of DASRRT*. DASRRT* has an average node count of 2479.27, which is significantly lower than all comparison algorithms, and the solving time is the shortest, with corresponding *p*-values all less than 0.05. This indicates that the proposed inefficient node optimization strategy effectively suppresses redundant node growth and improves solving speed. Regarding memory requirements, taking RRT* as an example, both RRT* and DASRRT* need to store node positions, parent node indices, and path costs, so the theoretical memory complexity is $O(N_{\mathrm{node}})$. DASRRT* introduces an additional inefficient node marking variable, slightly increasing memory usage per node. However, since the number of nodes is reduced by about 42.27%, the overall memory requirement is reduced. In terms of path smoothness, the average curvature and maximum turning angle of DASRRT* are significantly lower than those of all comparison algorithms, indicating that the proposed path optimization strategy effectively reduces the angularity and sharp turns in the sampled path, making the generated path more suitable as a reference trajectory for subsequent UAV formation tracking and cooperative control.

#### 5.1.2. Ablation Studies

To systematically evaluate the individual contributions and synergistic effects of the improved modules in the DASRRT* algorithm, comprehensive ablation experiments were conducted under the same simulation environment. All methods used identical parameter settings and were independently executed 50 times in the same scenario, and the averaged results are computed to mitigate the impact of randomness. For clarity, the following five modules are defined: A: baseline RRT* algorithm; B: hybrid guided sampling strategy; C: perception-based adaptive step size strategy; D: inefficient node optimization strategy; E: path optimization strategy.

**Figure 16:**
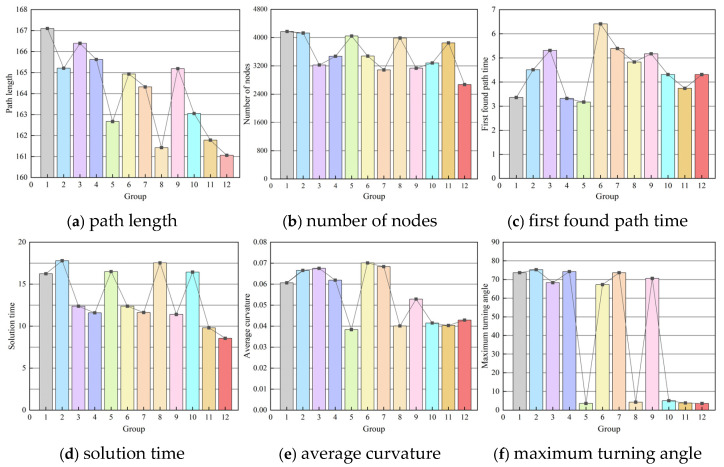
Comparison chart of ablation experiments.

Table 4 quantitatively compares the results of 12 ablation combinations, where Group 12 (A + B + C + D + E) corresponds to the complete DASRRT* algorithm. Figure 16 provides an intuitive illustration of the comparison results of 12 groups under different indicators.

By analyzing the results of the ablation experiment, using Group 1 as the reference, the incremental effects of each individual module are as follows:

Module B (Hybrid Guided Sampling): It provides some improvement in path length, but its main contribution lies in guiding the sampling points to focus on effective regions, laying the foundation for multi-module collaboration.

Module C (Perception-Adaptive Step Size): It shows good improvement in node count and solving time, indicating that this strategy effectively reduces invalid expansion in complex environments and improves search efficiency.

Module D (Inefficient Node Optimization): Significant improvements are seen in node count and solving time, validating the effectiveness of real-time redundant node pruning in suppressing the expansion of the search tree. This significantly reduces computational overhead while maintaining the path quality largely stable.

Module E (Path Optimization): It mainly affects the trajectory post-processing phase, with the most notable improvements in the maximum turning angle and average curvature, as well as further improvement in path length.

### 5.2. Simulation Validation of the Formation Control Algorithm

In this section, the proposed control algorithm is validated through simulations. The UAV formation consists of five UAVs, denoted as follower U1, follower U2, follower U3, follower U4, and follower U5. The virtual leader U0 flies along the trajectory planned by DASRRT*. U1 tracks the virtual leader, while the remaining followers track U1 and maintain a V-shaped formation with a rear included angle of $45^{\circ }$. The formation information is listed in Table 5, the parameters of the dynamic obstacles are given in Table 6.

**Figure 17:**
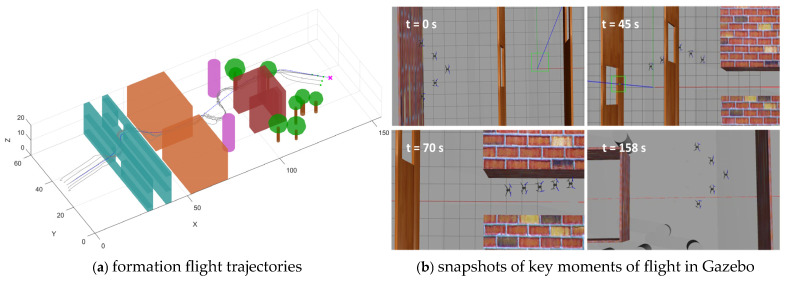
Formation flight validation results.

Figure 17a illustrate the flight trajectories of the UAV formation in three-dimensional space. Using the proposed algorithm, the UAVs successfully and safely traversed the complex environment, with a total flight time of 158 s. In the initial stage, the environment is relatively open, allowing the UAV swarm to maintain the V-shaped formation. Upon entering the obstacle region, under the dual-mode switching mechanism, each UAV responds rapidly to environmental changes and independently plans its obstacle-avoidance trajectory. After passing through the obstacle region, the UAVs quickly regain and maintain the desired V-shaped formation and reach the target point. The coordinated effect of the proposed dual-mode switching mechanism and the control law ensures both individual flight safety and formation integrity at the mission level. Figure 17b presents snapshots of key moments during the formation flight on the Gazebo platform. It can be seen that the UAV formation successfully completes takeoff, obstacle traversal, and arrival at the destination, indicating that the proposed method can be reproduced on the Gazebo platform. This further verifies the implementability of the algorithm and its potential for engineering applications.

**Figure 18:**
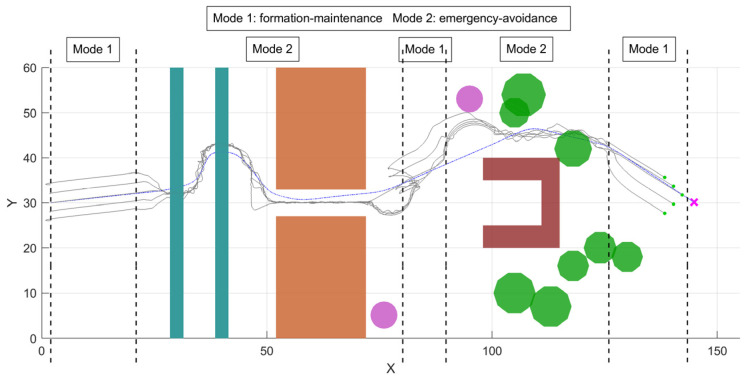
Top view of the formation flight trajectories.

**Figure 19:**
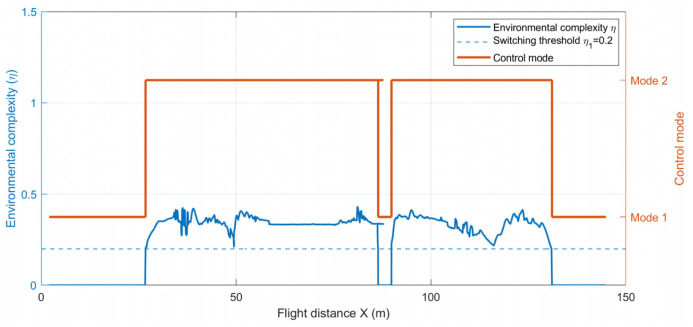
Mode switching and variations in environmental complexity.

**Figure 20:**
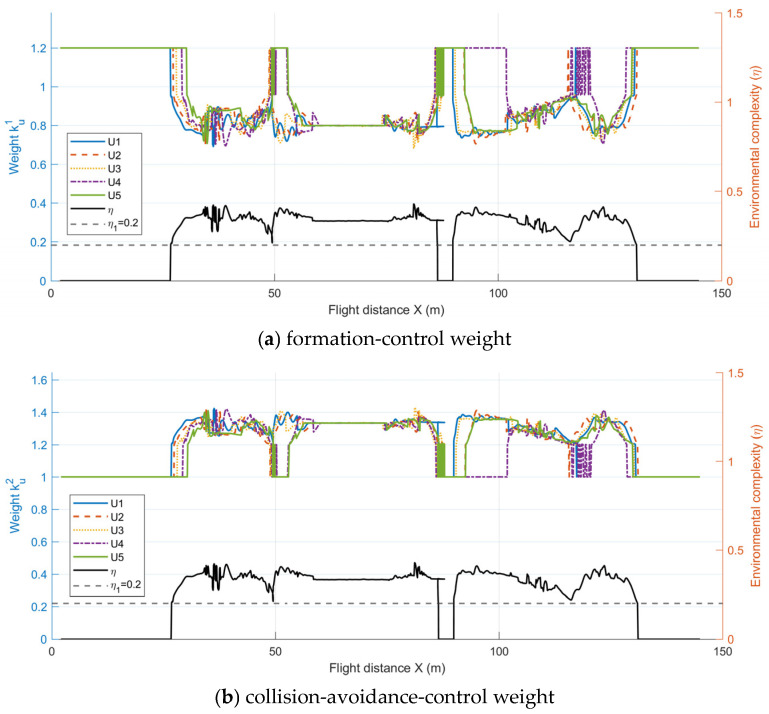

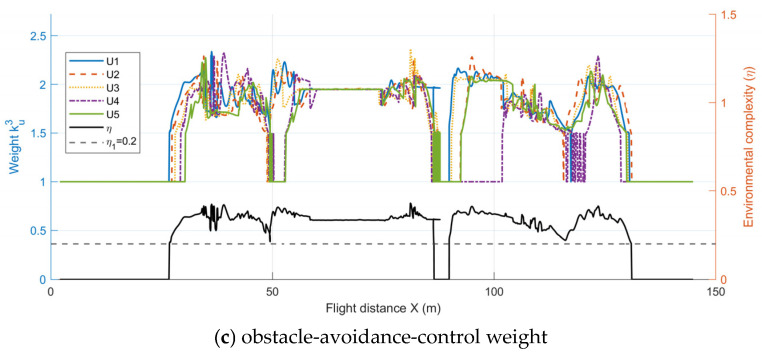
Variations in control-force weights and environmental complexity.

Figure 18, Figure 19 and Figure 20 illustrate the dynamic relationship among the environmental complexity η, mode switching, and the control-weight coefficients during flight. As shown in Figure 19, the formation flight undergoes two distinct transitions between obstacle avoidance and formation recovery. When the formation approaches a narrow passage or a densely cluttered mixed-obstacle region, the local environmental complexity increases and η exceeds the threshold, causing the system to switch from the formation maintenance mode to the Emergency-Avoidance Mode. After passing through the obstacle region, η falls below the threshold, and the system returns to the formation maintenance mode. As further illustrated in Figure 20, the variation in environmental complexity not only triggers mode switching, but also directly affects the allocation of control weights. In the formation maintenance mode, $k_{u}^{1}$ dominates, while $k_{u}^{2}$ and $k_{u}^{3}$ remain relatively small, so the system mainly focuses on maintaining the V-shaped formation. In the Emergency-Avoidance Mode, as η increases, $k_{u}^{1}$ decreases whereas $k_{u}^{2}$ and $k_{u}^{3}$ increase, with $k_{u}^{3}$ showing a more pronounced response in highly cluttered regions. This indicates that the control focus shifts from formation constraints to obstacle avoidance and collision avoidance. After clearing the obstacle region, the formation reconverges to the desired configuration. The results show that the designed dual-mode switching mechanism and dynamic weight adjustment strategy can autonomously adjust the control law weights based on environmental complexity, achieving an efficient balance between formation maintenance and flight safety.

The environmental complexity threshold is set based on multiple simulation experiments and an analysis of formation flight risk changes. To verify whether this switching strategy leads to frequent mode switching and control oscillations, the state changes during the flight were further statistically analyzed. Throughout the entire flight process, the system only experienced 4 mode switches, with an average of one switch every 39.4 s, and no high-frequency oscillation near the threshold was observed. The average formation recovery time was 2.8 s, and the maximum oscillation amplitude was 0.032946 m, indicating that the mode switching process did not cause significant control oscillations or flight instability. These results suggest that the set threshold effectively meets the stable switching requirements in this simulation scenario. In subsequent engineering deployments, the threshold switching logic can be further combined with a hysteresis interval to enhance robustness against sensor noise and transient disturbances.

**Figure 21:**
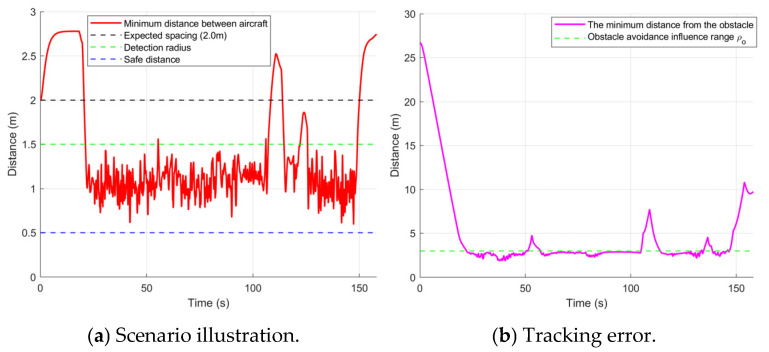
Analysis of the minimum safety distances during formation flight.

As shown in Figure 21a, within the obstacle region, the minimum inter-UAV distance remains around 1 m, and no inter-agent collisions occur throughout the flight. This verifies the effectiveness of the inter-UAV collision-avoidance control law. Figure 21b shows that when the UAVs enter the densely cluttered area, the minimum distance between the formation and obstacles stays at approximately 2.5 m and never drops to zero at any time. This indicates that the improved obstacle-avoidance control law—by incorporating a distance-adaptive factor and a dynamic velocity weighting—provides sufficient repulsive action to ensure safety while maintaining motion toward the goal.

**Figure 22:**
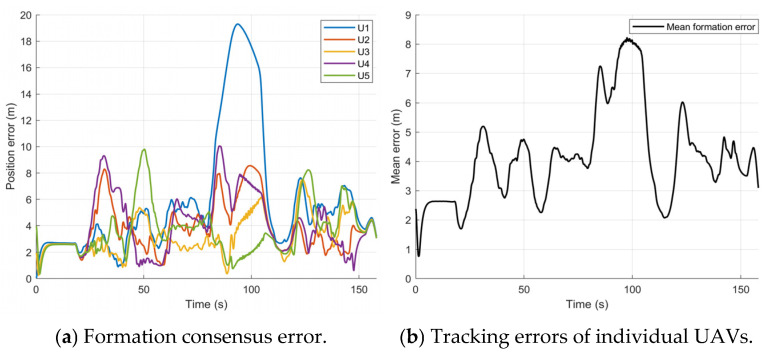
Error analysis of the formation control system.

**Figure 23:**
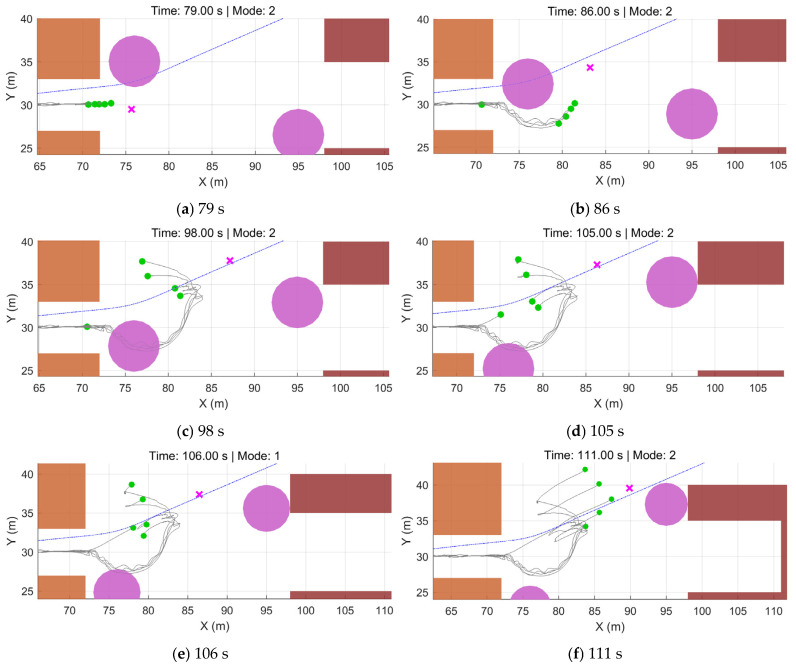
Snapshot of the formation during the waiting stage.

As shown in Figure 22, during the initial stage, the tracking errors of all UAVs rapidly converge to a small range near zero, verifying the fast convergence of the proposed control law. After entering the densely cluttered region, the system switches to the emergency-avoidance mode, and the priority on obstacle avoidance induces fluctuations in the tracking errors. Even during the narrow-passage traversal and dynamic avoidance phases, the mean error remains within a controlled range, while higher tracking accuracy is achieved in open areas. Notably, U1 exhibits a relatively large error around 90 s because it waits at the end of the narrow passage to avoid the first dynamic obstacle. The other UAVs perceive U1’s lag, move toward U1, and enter a waiting state. Once the dynamic obstacle passes, U1 immediately accelerates under the control input to catch up. Owing to the rapid convergence of the control law, the formation quickly returns to the prescribed configuration. The formation waiting stage is shown in Figure 23.

**Figure 24:**
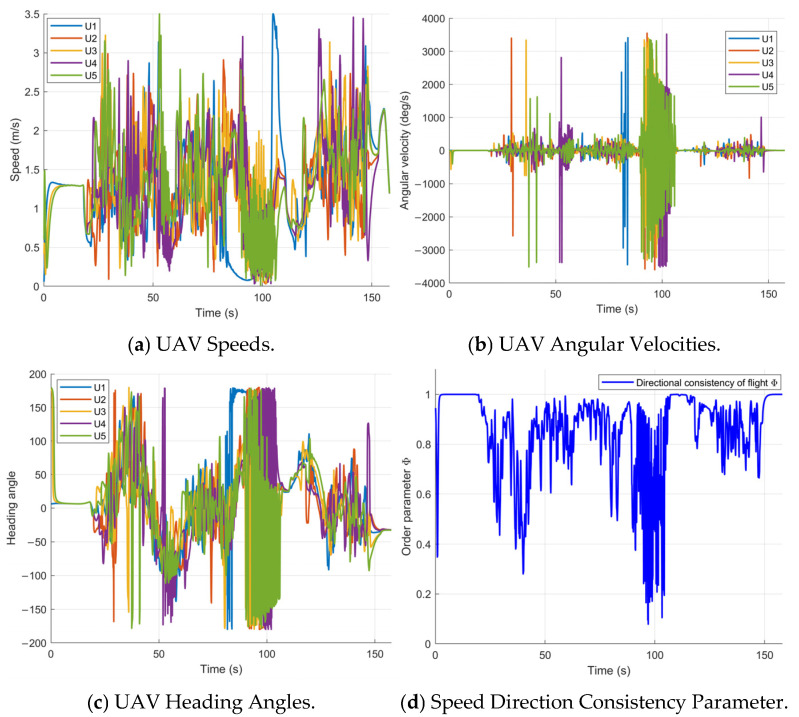
UAV flight data.

As shown in Figure 24a, the flight speed of all UAVs is strictly limited to a maximum of 3.5 m/s throughout the process. From Figure 24b,c, it can be observed that during the formation maintenance phase, the heading changes of all UAVs are generally consistent. However, during the obstacle-avoidance phase, both the heading angle and angular velocity show significant fluctuations, reflecting the UAVs’ rapid attitude adjustments to adapt to local obstacle constraints. The orderly parameter Φ is a core indicator of the degree of consistency in the formation’s speed and direction. As shown in Figure 24d, in the formation maintenance mode, Φ remains close to 1, indicating that the flight directions of all UAVs are highly consistent. In the obstacle-avoidance mode (e.g., around 18 s and 100 s), Φ significantly decreases, indicating that the UAVs have adopted differentiated avoidance actions. After the obstacle avoidance is completed, Φ quickly recovers to near 1, showing that the designed control framework has good cooperative recovery capability.

**Figure 25:**
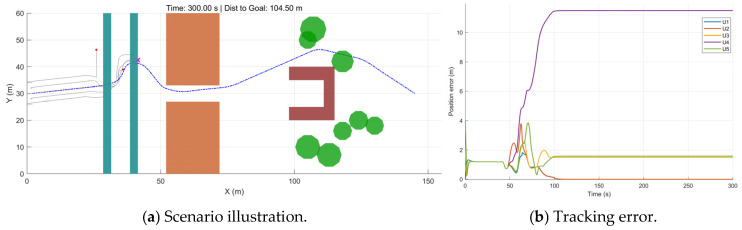
Experiment without the dual-mode switching mechanism.

As shown in Figure 25a, the UAV formation becomes stalled while attempting to traverse the obstacle region formed by narrow windows. From Figure 25b, U4 fails to pass through the window in time and consequently departs from the formation. After t≈100 s, all position errors converge to nearly constant values, indicating that the system enters a deadlock state: the resultant force becomes zero, causing the formation to halt. This demonstrates that, without the dual-mode switching mechanism, the cooperative control law alone cannot balance formation maintenance and obstacle avoidance in extremely constrained spaces.

To validate the effectiveness of the proposed improved APF in mitigating the issue of unreachable targets caused by local minima in the traditional APF, a standard APF control group was constructed, and a comparative experiment was conducted under the same conditions. This control group removed the dual-mode switching strategy, adaptive weight allocation, and the target distance and velocity coupling terms in the improved potential field, relying solely on the traditional APF repulsive force for external obstacle avoidance and inter-UAV collision avoidance.

**Figure 26:**
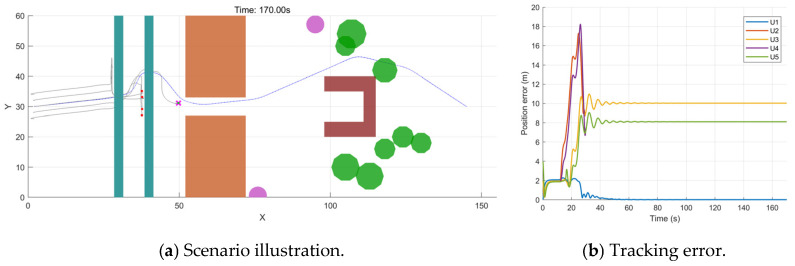
Formation flight experiment using the traditional APF.

As shown in Table 7 and Figure 26, although the APF does not lead to any collision, it fails to reach the target point and becomes trapped in the obstacle region, indicating that the traditional APF is prone to falling into local minima in complex obstacle environments. Its minimum obstacle distance is only 0.1187 m, and the average formation error is 6.9879 m, suggesting a low obstacle-avoidance safety margin and difficulty in balancing obstacle avoidance with formation maintenance. In contrast, the improved APF successfully reaches the target point, with the minimum obstacle distance increased to 1.9698 m and the average formation error reduced to 4.1844 m, indicating that the improved APF provides better obstacle-avoidance safety and formation maintenance capability in complex dynamic environments. The maximum oscillation amplitude of the improved APF is higher than that of the traditional APF, mainly because the improved APF successfully enters and traverses the subsequent narrow passage and dynamic-obstacle region, during which local trajectory variations become relatively larger due to obstacle avoidance. By contrast, the traditional APF becomes trapped before entering the subsequent obstacle region; therefore, the oscillation amplitudes of the two methods are not measured at the same motion stage, and a direct comparison is not entirely fair. The maximum oscillation amplitude of the improved APF remains within an acceptable range and does not cause collisions or mission failure, and can therefore be regarded as a necessary maneuvering response during complex obstacle avoidance. Overall, compared with the traditional APF, the improved APF demonstrates superior performance in task completion, local-minimum suppression, obstacle-avoidance safety margin, and formation maintenance capability.

To further verify the adaptability of the proposed hierarchical cooperative control framework under environmental disturbances and formation configuration changes, experiments are conducted from three aspects: initial disturbance, dynamic obstacle velocity variation, and formation scale variation. The results are shown in Table 8. Here, the average formation recovery time is defined as the average time it takes for the formation’s mean error to drop within the set threshold and remain there for several sampling steps after the system switches from Mode 2 to Mode 1.

In the initial disturbance experiment, Groups B and C achieved average formation errors of 3.5799 m and 3.703 m, respectively, both lower than the 4.15 m in the baseline scenario. The average recovery times were also shortened to 1.6 s and 0.9 s, indicating that the system can quickly absorb the formation deviation caused by the initial disturbance and restore cooperative flight. Although the minimum inter-UAV distances in both groups decreased, no collisions occurred, demonstrating that the controller maintains reliable safety constraints even under initial biases.

In the dynamic obstacle speed variation experiment, after adjusting the obstacle speed to 0.7 times and 1.3 times the baseline value in Groups D and E, the system still maintained zero collisions, with average formation errors further decreasing, and recovery times staying within a short range. In conditions with more frequent dynamic interactions, although the number of mode switches increased, the system was still able to quickly recover formation after safely avoiding obstacles. This indicates that the proposed dual-mode cooperative mechanism can adjust the control focus in response to time-varying environments and shows strong adaptability to changes in obstacle motion speed.

In the formation scale variation experiment, when the formation size was expanded from 3 UAVs to 7 and 9 UAVs, although the local interaction complexity increased significantly and both the minimum inter-UAV distance and minimum obstacle distance decreased, no collisions occurred in any of the scenarios. The average formation error and recovery time remained within acceptable ranges, demonstrating that the framework retains good task maintenance and cooperative recovery capabilities under scale variation conditions, reflecting a certain level of system scalability.

## 6. Conclusions

This paper addressed the coupling issues among global planning, local obstacle avoidance, and cooperative control for multi-UAV formations in complex urban dynamic environments. A hierarchical cooperative control framework based on a virtual leader–follower architecture was proposed. In the global planning layer, the DASRRT* algorithm was developed to overcome the limitations of traditional algorithms, including slow convergence, low expansion efficiency in narrow passages, and excessive turning angles in dense obstacle environments. The generated high-quality reference trajectories provided effective guidance for subsequent formation flight control. In the local obstacle avoidance layer, a distributed controller based on the improved APF method was designed to coordinate the control conflicts among inter-UAV collision avoidance, external obstacle avoidance, and formation maintenance. This controller improved formation consistency, stable tracking, and reconfiguration capability in complex dynamic environments. In the cooperative control layer, an environmental-complexity-based dual-mode switching mechanism was constructed to adaptively coordinate formation maintenance and emergency obstacle avoidance. Meanwhile, the real-time local target selection strategy enhanced tracking smoothness and robustness under complex path conditions. Simulation results show that DASRRT* achieved a maximum turning angle of only 5.11° and a final tree node count of 2479, representing an approximately 34–53% reduction compared with the other algorithms. These results indicate that the proposed method improved search efficiency and reduced the large attitude adjustments required to track paths with excessive turning angles. In complex urban scenarios with initial disturbances, obstacle speed changes, and formation scale variations, all test cases maintained collision-free operation, and the average formation error remained within 3.33–4.15 m. The average formation recovery time after mode switching was also relatively short. These results demonstrate that the proposed hierarchical cooperative control framework achieved strong robustness, environmental adaptability, and scalability.

Future work will focus on the real-time onboard implementation of the proposed framework in complex scenarios. In particular, computational efficiency, communication delay, sensor uncertainty, and hardware resource constraints will be further considered to improve the feasibility of deploying the method in real multi-UAV systems. In addition, learning-based alternatives, such as deep reinforcement learning methods for dynamic obstacle avoidance and adaptive decision-making, will be further investigated and compared with the proposed framework to evaluate their respective advantages in complex and uncertain environments.

## Figures and Tables

**Table 1:** Core parameters specific to the algorithms.

Algorithm	Specific Parameters
RRT*	No additional parameters
Informed RRT*	$P_{g}=0.3,$ $\;\gamma_{IRRT*}=1.1$
RRT*_APF	$k_{APF}=1.5,$ $\;r_{u}=8\;m$ $,\;\alpha_{APF}=0.3$
RRT*_PRIME	$w_{1}=0.5,w_{2}=0.2,w_{3}=0.3,P_{tar}=0.6,K_{cand}=10,\theta_{bias}=\pi /3,$ $w_{4}=2,w_{5}=3,w_{6}=1,C_{th}=20,E_{th}=60,\;\;\gamma_{1}=0.6,\gamma_{2}=0.4,\kappa =5$
BIT*	$N_{s}=7000$ $,\;N_{b}=150,$ $\eta_{BIT}=1.3$ $,\;r_{min}=2\;m$ $,\;r_{max}=20\;m$
DASRRT*	$p_{f}=0.3$ $,P_{g}=0.3,\;\rho =1,\;\;\lambda =\frac{1}{3},\beta =0.8,\;\Delta s_{smooth}=0.2\;m,\;\delta_{min}=0.4\;m$

**Table 2:** Performance statistics for different path planning algorithms.

Indicators		RRT*	Informed RRT*	RRT*_APF	RRT*_PRIME	BIT*	DASRRT*
Path length (m)	mean	167.04	165.54	164.16	158.33	163.35	161.13
	Standard deviation	2.48	1.90	2.60	1.47	2.31	2.74
	Confidence interval	[165.67, 168.42]	[164.49, 166.59]	[162.72, 165.60]	[157.51, 159.14]	[162.07, 164.63]	[159.62, 162.65]
Number of nodes	mean	4295.07	3796.87	4511.60	5276.53	2879.13	2479.27
	Standard deviation	482.69	587.53	434.90	238.23	100.57	275.51
	Confidence interval	[4027.76, 4562.37]	[3471.51, 4122.23]	[4270.76, 4752.44]	[5144.61, 5408.46]	[2823.44, 2934.83]	[2326.70, 2631.84]
Solution time (s)	mean	17.41	12.72	23.39	49.83	24.45	8.03
	Standard deviation	2.36	2.06	4.20	5.51	2.30	0.78
	Confidence interval	[16.10, 18.71]	[11.58, 13.86]	[21.06, 25.72]	[46.78, 52.88]	[23.18, 25.73]	[7.60, 8.46]
$\mathrm{Mean}\;\mathrm{curvature}\;(m^{-1}$)	mean	0.0649	0.0709	0.0575	0.0570	0.0513	0.0403
	Standard deviation	0.0134	0.0185	0.0069	0.0109	0.0056	0.0028
	Confidence interval	[0.0575, 0.0723]	[0.0606, 0.0811]	[0.0537, 0.0613]	[0.0510, 0.0631]	[0.0482, 0.0544]	[0.0388, 0.0419]
Maximum turning angle (°)	mean	72.09	76.22	79.11	71.90	79.49	5.11
	Standard deviation	12.79	14.24	16.21	11.23	15.94	1.84
	Confidence interval	[65.01, 79.18]	[68.33, 84.10]	[70.14, 88.09]	[65.68, 78.12]	[70.66, 88.32]	[4.09, 6.13]

**Table 3:** *p*-values of the t-test between DASRRT* and comparison algorithms.

Comparison Algorithms	Path Length (m)	Number of Nodes	Solution Time (s)	Mean Curvature (m^−1^)	Maximum Turning Angle (°)
RRT*	1.48 × 10^−5^	2.71 × 10^−9^	1.49 × 10^−9^	9.13 × 10^−6^	7.21 × 10^−12^
Informed RRT*	1 × 10^−4^	3.36 × 10^−7^	2.18 × 10^−6^	3.17 × 10^−5^	1.96 × 10^−11^
RRT*_APF	0.021	5.47 × 10^−11^	1.51 × 10^−9^	4.31 × 10^−7^	7.77 × 10^−11^
RRT*_PRIME	0.0029	7.04 × 10^−13^	6.22 × 10^−14^	1.09 × 10^−4^	4.17 × 10^−12^
BIT*	0.058	1.48 × 10^−4^	1.41 × 10^−14^	7.97 × 10^−6^	4.46 × 10^−11^

**Table 4:** Ablation experiment results.

Group	Included Modules	Path Length (m)	Number of Nodes	First Found Path Time (s)	Solution Time (s)	Average Curvature	Maximum Turning Angle (°)
Group 1	A	167.1	4173	3.36	16.22	0.0606	73.56
Group 2	A + B	165.21	4128	4.51	17.79	0.0665	75.18
Group 3	A + C	166.39	3226	5.31	12.37	0.0675	68.28
Group 4	A + D	165.62	3473	3.32	11.57	0.0619	74.18
Group 5	A + E	162.67	4044	3.17	16.48	0.0383	3.59
Group 6	A + B + C	164.93	3477	6.41	12.37	0.0701	67.21
Group 7	A + B + D	164.32	3086	5.39	11.61	0.0683	73.55
Group 8	A + B + E	161.43	3987	4.83	17.53	0.0401	4.29
Group 9	A + C + D	165.19	3135	5.17	11.39	0.0529	70.59
Group 10	A + C + E	163.05	3283	4.31	16.42	0.0415	5.09
Group 11	A + D + E	161.78	3850	3.74	9.79	0.0403	3.87
Group 12	A + B + C + D + E	161.06	2672	4.31	8.56	0.0389	3.62

**Table 5:** Formation information of the UAV swarm.

Parameter	Value	Parameter	Value
Number of UAVs	5	Initial position of U3	[2, 28, 5]
Maximum flight speed	3.5	Initial position of U4	[2, 34, 5]
Maximum acceleration	6.0	Initial position of U5	[2, 26, 5]
Desired spacing	2.0	Desired relative offset of U2 w.r.t. U1	[−2, 2, 0]
UAV sensing radius	5	Desired relative offset of U3 w.r.t. U1	[−2, −2, 0]
Initial position of U1	[2, 30, 5]	Desired relative offset of U4 w.r.t. U1	[−4, 4, 0]
Initial position of U2	[2, 32, 5]	Desired relative offset of U5 w.r.t. U1	[−4, −4, 0]

**Table 6:** Information of dynamic obstacles.

Obstacle	Initial Center Coordinate (m)	Radius (m)	Height (m)	Velocity (m/s)
Dynamic obstacle 1	[82.8, 65, 10]	3	20	[0, −0.325, 0]
Dynamic obstacle 2	[95, −5, 10]	3	20	[0, 0.4, 0]

**Table 7:** Quantitative comparison results of APF and improved APF.

Method	Target Reached	Collision	Minimum Obstacle Distance (m)	Average Formation Error (m)	Trapped in Local Minima	Maximum Oscillation Amplitude (m)
APF	×	×	0.1187	6.9879	√	0.0069
Improved APF	√	×	1.9698	4.1844	×	0.0329

**Table 8:** Adaptability verification results of the control algorithm under different environmental disturbances and formation configurations.

Group	Change Type	Min UAV Distance (m)	Min Obstacle Distance (m)	Average Formation Error (m)	Average Formation Recovery Time (s)	Mode Switch Count
A	standard scenario	0.5976	1.8961	4.15	3.3	4
B	initial disturbance in y (+5 m)	0.4223	1.6595	3.5799	1.6	4
C	initial disturbance in y (−5 m)	0.5271	1.4794	3.703	0.9	4
D	obstacle speed ×0.7	0.6162	1.8961	3.4912	0.5667	18
E	obstacle speed ×1.3	0.6162	1.8961	3.3274	0.6	4
F	3 UAV formation	0.8257	2.1074	3.675	2	4
G	7 UAV formation	0.3811	1.0905	3.5428	0.8	8
H	9 UAV formation	0.4169	1.3338	3.5908	0.75	4

## Data Availability

Dataset available on request from the authors.

## References

[1] Chung S.-J., Paranjape A.A., Dames P., Shen S., Kumar V. (2018). A Survey on Aerial Swarm Robotics. IEEE Trans. Robot..

[2] Song J., Zhao K., Liu Y. (2023). Survey on Mission Planning of Multiple Unmanned Aerial Vehicles. Aerospace.

[3] Wang X., Zhao Z., Yi L., Ning Z., Guo L., Yu F.R., Guo S. (2025). A Survey on Security of UAV Swarm Networks: Attacks and Countermeasures. ACM Comput. Surv..

[4] Zheng D., Zhang Y., Li F., Cheng P. (2023). UAVs Cooperative Task Assignment and Trajectory Optimization with Safety and Time Constraints. Def. Technol..

[5] Gu Y., Guo K., Guo L., Qiao J., Jia J., Yu X., Xie L. (2021). An Enhanced UAV Safety Control Scheme against Attacks on Desired Trajectory. Aerosp. Sci. Technol..

[6] Halder S., Ghosal A., Conti M. (2023). Dynamic Super Round-Based Distributed Task Scheduling for UAV Networks. IEEE Trans. Wirel. Commun..

[7] Rigatos G., Abbaszadeh M., Siano P., AL-Numay M., Cuccurullo G., Gao Z. (2025). Nonlinear optimal and multi-loop flatness-based control for dual-UAV cooperative load transportation. J. Field Robot..

[8] Wang Z., Hu T., Long L. (2023). Multi-UAV Safe Collaborative Transportation Based on Adaptive Control Barrier Function. IEEE Trans. Syst. Man Cybern. Syst..

[9] Han D., Jiang H., Wang L., Zhu X., Chen Y., Yu Q. (2024). Collaborative Task Allocation and Optimization Solution for Unmanned Aerial Vehicles in Search and Rescue. Drones.

[10] Wu J., Luo C., Min G., McClean S. (2024). Formation Control Algorithms for Multi-UAV Systems with Unstable Topologies and Hybrid Delays. IEEE Trans. Veh. Technol..

[11] Debnath D., Vanegas F., Sandino J., Hawary A.F., Gonzalez F. (2024). A Review of UAV Path-Planning Algorithms and Obstacle Avoidance Methods for Remote Sensing Applications. Remote Sens..

[12] Gao J., Pan W. (2024). Research, Analysis, and Improvement of Unmanned Aerial Vehicle Path Planning Algorithms in Urban Ultra-Low Altitude Airspace. Aerospace.

[13] Zheng Z., Huang H., Li C., Yu Y., Wang X., Cai J., Huang X., Hu S. (2025). A Hybrid Search Behavior-Based Adaptive Grey Wolf Optimizer for Cooperative Path Planning for Multiple UAVs. Sensors.

[14] Merei A., Mcheick H., Ghaddar A., Rebaine D. (2025). A Survey on Obstacle Detection and Avoidance Methods for UAVs. Drones.

[15] Ge G., Sun M., Xue Y., Pavlova S. (2025). Transformer-Based Soft Actor–Critic for UAV Path Planning in Precision Agriculture IoT Networks. Sensors.

[16] Bai X., Jiang H., Cui J., Lu K., Chen P., Zhang M. (2021). UAV Path Planning Based on Improved A∗ and DWA Algorithms. Int. J. Aerosp. Eng..

[17] Xu H., Niu Z., Jiang B., Zhang Y., Chen S., Li Z., Gao M., Zhu M. (2024). ERRT-GA: Expert Genetic Algorithm with Rapidly Exploring Random Tree Initialization for Multi-UAV Path Planning. Drones.

[18] Yu J., Piao H., Hou Y., Mo L., Yang X., Zhou D. (2022). DOMA: Deep Smooth Trajectory Generation Learning for Real-Time UAV Motion Planning. Proc. Int. Conf. Autom. Plan. Sched..

[19] Lindqvist B., Patel A., Löfgren K., Nikolakopoulos G. (2024). A Tree-Based Next-Best-Trajectory Method for 3-D UAV Exploration. IEEE Trans. Robot..

[20] Peng Y., Zhang X., Xie H., Xu X. (2025). Hybrid Distributed and Decentralised Reinforcement Learning for Formation Control of Multi-Robots with Obstacle Avoidance. CAAI Trans. Intell. Technol..

[21] Guerrero-Castellanos J.F., Vega-Alonzo A., Durand S., Marchand N., Gonzalez-Diaz V.R., Castañeda-Camacho J., Guerrero-Sánchez W.F. (2019). Leader-Following Consensus and Formation Control of VTOL-UAVs with Event-Triggered Communications. Sensors.

[22] Petracek P., Walter V., Baca T., Saska M. (2021). Bio-Inspired Compact Swarms of Unmanned Aerial Vehicles without Communication and External Localization. Bioinspir. Biomim..

[23] Ding W., Zhang L., Zhang G., Wang C., Chai Y., Yang T., Mao Z. (2024). Research on Obstacle Avoidance of Multi-AUV Cluster Formation Based on Virtual Structure and Artificial Potential Field Method^★^. Comput. Electr. Eng..

[24] Sheng H., Zhang J., Yan Z., Yin B., Liu S., Bai T., Wang D. (2023). New multi-UAV formation keeping method based on improved artificial potential field. Chin. J. Aeronaut..

[25] Zhang T., Dong D., Du Z., Long J., Yu D., Wang Z., Chen C.L.P. (2023). Swarm Control Based on Artificial Potential Field Method with Predicted State and Input Threshold. Eng. Appl. Artif. Intell..

[26] Fan J., Chen X., Liang X. (2023). UAV Trajectory Planning Based on Bi-Directional APF-RRT* Algorithm with Goal-Biased. Expert Syst. Appl..

[27] Zheng Y., Li A., Chen Z., Wang Y., Yang X., Im S.-K. (2025). MPN-RRT*: A New Method in 3D Urban Path Planning for UAV Integrating Deep Learning and Sampling Optimization. Sensors.

[28] Guo J., Xia W., Hu X., Ma H. (2022). Feedback RRT* Algorithm for UAV Path Planning in a Hostile Environment. Comput. Ind. Eng..

[29] Fan J., Chen X., Wang Y., Chen X. (2022). UAV Trajectory Planning in Cluttered Environments Based on PF-RRT* Algorithm with Goal-Biased Strategy. Eng. Appl. Artif. Intell..

[30] Zhang W., Xu G., Song Y., Wang Y. (2023). An Obstacle Avoidance Strategy for Complex Obstacles Based on Artificial Potential Field Method. J. Field Robot..

[31] Zhang P., He Y., Wang Z., Li S., Liang Q. (2024). Research on Multi-UAV Obstacle Avoidance with Optimal Consensus Control and Improved APF. Drones.

[32] Guo J., Qi J., Wang M., Wu C., Ping Y., Li S., Jin J. (2023). Distributed Cooperative Obstacle Avoidance and Formation Reconfiguration for Multiple Quadrotors: Theory and Experiment. Aerosp. Sci. Technol..

[33] Yan Z., Zhang C., Zhang K., Cai S. (2023). Research on Guaranteed Cost Formation Tracking Control of Multi-AUV with Virtual Leader. Proceedings of the OCEANS 2023-Limerick, Limerick, Ireland, 5–8 June 2023.

[34] Liu Y., Chen C., Wang Y., Zhang T., Gong Y. (2024). A Fast Formation Obstacle Avoidance Algorithm for Clustered UAVs Based on Artificial Potential Field. Aerosp. Sci. Technol..

[35] Wang X., Ye D., Wei F. (2024). Optimized Bipartite Formation Control for Multiagent Systems with Obstacle and Collision Avoidance. Inf. Sci..

[36] Li Y., Zhang P., Wang Z., Rong D., Niu M., Liu C. (2024). Multi-UAV Obstacle Avoidance and Formation Control in Unknown Environments. Drones.

[37] Shao S., Zhang J., Wang T., Shankar A., Maple C. (2024). Dynamic Obstacle-Avoidance Algorithm for Multi-Robot Flocking Based on Improved Artificial Potential Field. IEEE Trans. Consum. Electron..

[38] Du Z., Zhang H., Wang Z., Yan H. (2024). Model Predictive Formation Tracking-Containment Control for Multi-UAVs with Obstacle Avoidance. IEEE Trans. Syst. Man Cybern. Syst..

[39] Khial N., Mhaisen N., Ismail L., Mabrok M., Mohamed A. (2025). Multi-Target Path Planning with Probabilistic Detection in Cluttered Environments. Proceedings of the ICC 2025-IEEE International Conference on Communications, Montreal, QC, Canada, 8–12 June 2025.

[40] Shevitz D., Paden B. (1994). Lyapunov stability theory of nonsmooth systems. IEEE Trans. Autom. Control.

[41] Gammell J.D., Srinivasa S.S., Barfoot T.D. (2014). Informed RRT*: Optimal Sampling-Based Path Planning Focused via Direct Sampling of an Admissible Ellipsoidal Heuristic. Proceedings of the 2014 IEEE/RSJ International Conference on Intelligent Robots and Systems, Chicago, IL, USA, 14–18 September 2014.

[42] Yan Y., Liu S., Hao R. (2025). RRT*-APF Path Planning and MA-AADRC-SMC Control for Cooperative 3-D Obstacle Avoidance in Multi-UAV Formations. Drones.

[43] Miao Y., Liu H., Zhang Z., Liang Y. (2025). Leveraging RRT*: Probabilistically Interpreted Mechanisms Enhanced with P-HOPE and FLEX-OPT for Complex Path Planning. IEEE Access.

